# Advances in Porous Silicon Materials for Sensing, Energy Storage, and Microelectronics

**DOI:** 10.3390/nano16040257

**Published:** 2026-02-15

**Authors:** Yujie Wang, Donghua Wang

**Affiliations:** 1 School of Electronics and Information Engineer, Hangzhou Dianzi University, Hangzhou 310018, China; 23041401@hdu.edu.cn; 2 School of Electronics and Information & Institute of Carbon Neutrality and New Energy, Hangzhou Dianzi University, Hangzhou 310018, China

**Keywords:** porous silicon, metal-assisted chemical etching, surface modification, optical biosensing, electrochemical energy storage

## Abstract

Porous silicon (PSi), characterized by its high specific surface area and highly tunable morphology, presents significant potential across optoelectronics, energy storage, and biomedical applications. This review provides a systematic analysis of the synthesis methodologies, interfacial chemical engineering, and diverse applications of PSi. Initially, fabrication techniques are examined, contrasting the pore formation mechanisms of electrochemical anodization, metal-assisted chemical etching (MACE), and emerging vapor-phase etching methods, while elucidating the control of geometric parameters from microporous to macroporous scales. To address the thermodynamic instability of the hydride-terminated surface, this review systematically evaluates modification strategies such as thermal oxidation, hydrosilylation, carbonization, and atomic layer deposition (ALD). We critically analyze their efficacy in mitigating oxidative drift and enabling specific functionalization. Subsequently, the review summarizes current applications in sensing (refractive index and photoluminescence modulation), energy storage (lithium-ion battery anodes and supercapacitors), and microsystem technologies (radio frequency (RF) isolation, gettering, and micro-electro-mechanical systems (MEMS) sacrificial layers), emphasizing the critical role of structure–property relationships. Finally, an objective assessment is provided regarding the challenges in translating PSi technology to industrial scales, specifically addressing the trade-offs between biodegradability and stability, wafer-scale process uniformity, and the compatibility of wet-chemical processing with standard complementary metal–oxide–semiconductor (CMOS) integration flows.

## 1. Introduction

Porous silicon (PSi) refers to a class of silicon-based porous architectures produced by controlled electrochemical or chemical dissolution, yielding interconnected pore networks within a crystalline silicon framework. Early observations of silicon electrochemical dissolution and surface restructuring were reported in the mid-20th century, most notably in the context of electrolytic shaping [1]. For several decades, PSi was largely treated as an etching-related morphology rather than a functional materials platform. Interest expanded substantially after the demonstration of room-temperature visible photoluminescence from electrochemically prepared porous layers [2], which catalyzed sustained research into quantum confinement, surface states, and nanostructure-enabled optical responses. Since then, PSi has transitioned from a photoluminescent curiosity to a broadly used scaffold whose properties can be tuned through pore geometry, backbone crystallinity, and surface termination [3,4,5].

A consistent description of PSi requires explicit attention to pore size and hierarchy. Following IUPAC conventions, pores are commonly classified as micropores (<2 nm), mesopores (2–50 nm), and macropores (>50 nm) [6]. In practice, many PSi films exhibit distributions across these regimes, including gradient or multiscale porosity, such that “micro/meso/macro” designations often describe the dominant pore width rather than a single-valued structure. Beyond pore width, porosity (void fraction), layer thickness, tortuosity/connectivity, and surface chemistry jointly determine mass transport, optical effective refractive index, mechanical integrity, and electrochemical accessibility—parameters that directly map onto device performance in sensing, energy storage, and microfabrication contexts.

PSi remains actively investigated because its structural and chemical degrees of freedom enable application-specific optimization while retaining compatibility with silicon manufacturing. High internal surface area supports adsorption- and binding-driven transduction in chemical and biosensing, and tunable optical thickness enables interferometric and photonic designs (e.g., microcavities and Bragg/Rugate structures). At the same time, the as-prepared surface is often hydrogen-terminated and susceptible to oxidation and property drift, making stabilization and functionalization central to reproducible performance across environments [7,8]. In energy storage, porous architectures can accommodate silicon’s large lithiation-induced volume changes, but the accompanying increase in interfacial area introduces competing effects such as enhanced SEI formation and reduced volumetric energy density, motivating composite and coating strategies [9,10]. In microelectronics and MEMS, oxidized or modified PSi offers routes to low-loss isolation, gettering, sacrificial layers, and high-density passive components, yet integration constraints (wet processing, contamination control, and thermal budgets) remain nontrivial [11,12]. Distinct from prior reviews that predominantly focus on isolated application domains or specific fabrication parameters, this work bridges the gap between emerging synthesis protocols (e.g., vapor-phase etching, substrate-native micromachining) and system-level integration constraints. In this Review, we therefore focus on how synthesis routes (anodization, MACE, and emerging methods) define morphology, how surface chemistry governs stability and interface function, and how these coupled structure–surface relationships translate into performance and trade-offs across sensing, energy storage, and silicon-based microsystems.

## 2. Synthesis and Morphological Control

### 2.1. Electrochemical Anodization

Electrochemical anodization remains the cornerstone of porous silicon (PSi) fabrication, valued for its ability to precisely tailor pore morphology through kinetic control. As comprehensively reviewed by Föll et al. [13], the fundamental dissolution mechanism in HF-based electrolytes relies on the localized flow of holes (h+) to the silicon surface, where the competition between oxide formation and direct dissolution dictates the pore geometry. Current density serves as the primary parameter governing these kinetics. According to the foundational models described by Sailor [3], increasing the current density typically accelerates the etching rate and enlarges pore diameter within the electropolishing limit. This kinetic principle has been rigorously validated across various substrates; for instance, Pratama et al. [14] reported a direct positive correlation between current densities (varied from 10 to 50 mA/cm^2^) and both pore depth and diameter on n-type Si (100) and (111) substrates. Extending this to p-type substrates, Mebed et al. [15] confirmed a similar linear relationship in highly doped silicon and further emphasized that careful adjustment of the anodization potential is crucial for balancing pore formation versus dissolution pathways, particularly for applications requiring strict size control such as filtration or microfluidics.

Beyond electrical parameters, the composition of the electrolyte—specifically the inclusion of surfactants—plays a decisive role in defining structural uniformity and dimensions. Sailor [3] highlights that ethanol is essential for reducing surface tension and facilitating the removal of hydrogen bubbles, a theoretical prerequisite for uniform etching. Building on this, Suryana et al. [16] systematically investigated HF:ethanol volume ratios (1:1, 1:3, and 3:1), demonstrating that higher ethanol content effectively modulates HF distribution at the interface, thereby yielding more uniform pore size distributions. Complementing uniformity control, Burham et al. [17] utilized solvent composition as a tuning knob for pore size; by varying HF:ethanol ratios from 3:7 to 9:1 under a fixed current density of 25 mA/cm^2^, they demonstrated that pore diameter could be tuned over a significant range, establishing solvent ratio as a critical variable alongside current density.

Finally, the pore formation mechanism is intrinsically linked to the doping type of the substrate. While p-type silicon etches readily under forward bias, n-type silicon typically requires backside illumination to generate the necessary holes. However, establishing a route for processing low-doped substrates without illumination, Lehmann and Gösele [18] originally identified that macroporous structures can form on n-type silicon in the dark via a breakdown mechanism. Leveraging this fundamental insight, Fulton et al. [19] successfully fabricated high-aspect-ratio macropores with a mesoporous transition layer on low-doped n-Si via dark anodization. This work confirms that by precisely tuning the anodization conditions to trigger avalanche breakdown, electrochemical anodization can be robustly extended to generate well-defined macroporous architectures even in the absence of external light sources.

Taken together, these experimental results reinforce the basic anodization principles: (1) increasing current density and anodization time generally increases pore depth and overall layer thickness; (2) solvent composition such as ethanol content plays a decisive role in controlling pore uniformity and size distribution; and (3) even low-doped or varied crystalline orientations of silicon can yield distinct porous morphologies through appropriate parameter control. This body of work shows that electrochemical anodization remains a robust and tunable platform for generating porous silicon with pore structures ranging from nanoporous to macroporous scales for applications in sensing, optoelectronics, and energy materials.

### 2.2. Metal-Assisted Chemical Etching

Metal-assisted chemical etching (MACE) is generally characterized as a localized electrochemical–chemical coupling process induced by metal catalysts within an HF/oxidant system. A consistent mechanistic description posits that metals (e.g., Au, Ag, Pt, Pd) catalyze the reduction of oxidants (commonly H_2_O_2_) in solution, functioning as localized “cathodes.” Simultaneously, holes (h^+^) are generated and injected into the silicon at the metal/silicon interface—acting as the localized “anode”—thereby driving silicon oxidation and subsequent dissolution by HF. This framework is frequently described as “wireless, short-circuited microscopic galvanic cells,” wherein interfacial redox processes occur simultaneously and self-consistently within the Si–metal–oxidant nanosystem. In a typical two-step MACE process, H_2_O_2_ reduction on the metal generates holes that traverse the catalyst to inject into the silicon valence band; the silicon is then oxidized and dissolved as fluorosilicate complexes at the interface (e.g., Si + 4h^+^ + 6HF → SiF_6_^2−^ + 6H^+^). This strict confinement of the reaction to the Si/metal interface elucidates the “transcription” characteristic, where the etching front spatially follows the metal pattern [20].

Building upon the feature that the metal pattern defines the reaction front and drives anisotropic propagation, a direct reason for MACE’s process flexibility in device-level transcription lies in its electroless attribute. As highlighted by Li et al., this process does not rely on an external power source or counter electrode to close the macroscopic current loop, thereby reducing dependence on the sample’s overall conductive pathway [21]. From a fabrication perspective, this “no counter electrode/no external bias” characteristic lowers requirements for backside ohmic contacts and electrode fixturing, allowing controlled etching in patterned structures with limited electrical contact. For instance, Akan et al. utilized wet MACE to transfer Au zone plate patterns into Si for hard X-ray Pd/Si zone plates. They reported an etching rate of 700 nm·min^−1^ for structures with a 30 nm outer width and ~900 nm thickness, corresponding to an aspect ratio of ~30:1. The authors noted that while electrodeposition into such deep, narrow Si molds is restricted by difficulties in establishing electrical contact at the bottom Au layer, electroless metal deposition combined with MACE effectively circumvents these contact issues [22].

This same low dependence on macroscopic conductive loops opens process windows for pursuing extreme aspect ratios under varying phase conditions. Romano et al. reported Pt-assisted gas-phase MacEtch, achieving vertical nanowires with lengths on the order of hundreds of micrometers within a 10–100 nm feature size range. They demonstrated a maximum etch depth of 107 μm and a peak rate of 25 μm·h^−1^, describing aspect ratios reaching 10,000:1 with high-fidelity pattern transfer at the 10 nm scale [23]. These results illustrate that without introducing external circuits, MACE can cover a morphology window ranging from the nanoscale to the microscale through the regulation of catalyst stability and interfacial mass transport.

In liquid-phase systems, wafer-scale achievements have further demonstrated this capability; Zhang et al. fabricated micro-pillar arrays on 4-inch wafers with an AR > 140:1 and depths reaching 285 μm. Furthermore, by optimizing the etching ratio and incorporating thermal oxidation treatment, the average sidewall RMS roughness was reduced from 42.3 nm to 15.8 nm. Expanding beyond conventional photolithography, novel cost-effective patterning methods such as electrospinning are increasing structural versatility [24]. In this approach, electrospun palladium (Pd) nanoclusters with sub-micron linewidths serve as tunable masks, acting as catalysts in acidic conditions to form semicircular structures, or as protective masks in alkaline environments to create pyramid-shaped microgrooves with 54.74° crystallographic angles [25].

**Figure 1:**
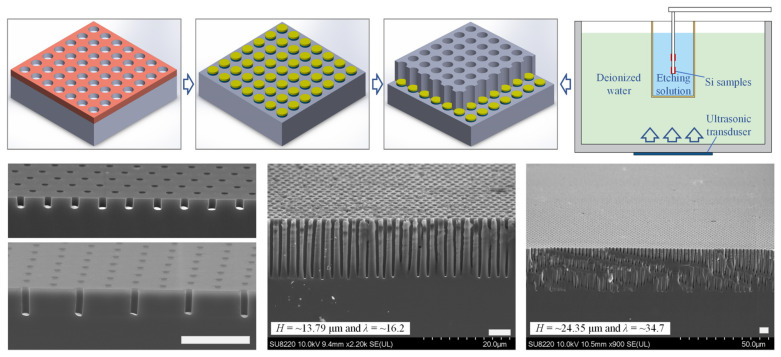
Ultrasound-enhanced mass transport in metal-assisted chemical etching (MACE). (**Top**) Schematic setup where ultrasonic waves induce microstreaming to replenish reactants at the etch front. (**Bottom**) Cross-sectional SEM images confirming the formation of uniform, high-aspect-ratio (>30:1) silicon nanohole arrays, demonstrating that physical agitation effectively overcomes diffusion limitations in deep etching [29].

However, despite these capabilities, as targets shift from nanostructures to “deep micro-structure etching” in liquid media, mass transport limitations (reactant supply/product removal) become a critical constraint [26]. To address this, strategies ranging from solvent engineering to physical field enhancement have been developed. Nur’aini and Oh utilized “ultra-thin metal films” to promote out-of-plane mass transport [27], while Akan and Vogt introduced isopropanol to optimize kinetics [28]. Figure 1 illustrates a pioneering ultrasound-enhanced MACE strategy reported by Long et al., designed to physically overcome these diffusion limits. As shown in the schematic (top panel, Figure 1), the silicon substrate with a patterned metal catalyst (yellow) is immersed in an etching solution, which is coupled to an ultrasonic transducer in a water bath. The induced ultrasonic waves generate convection and microstreaming flows that actively replenish reactants at the etch front. This enhancement results in a maximum etching rate of ∼0.82 μm·min^−1^ (22.73% higher than conventional MACE). The corresponding cross-sectional SEM images (bottom panel, Figure 1) confirm the fabrication of large-area, uniform Si nanohole arrays with depths (H) reaching ∼24.35 μm and aspect ratios (λ) of ∼34.8, demonstrating unprecedented verticality and uniformity [29]. Complementing physical field enhancement, dynamic fluid regulation has also proven effective. Recent molecular dynamics studies have highlighted that modulating the interfacial shear of nanoconfined hydration layers via surface charging can fundamentally alter mass transport resistance in narrow channels [30]. Drawing on these principles, a recently established concentration-regulated MACE system, validated by CFD simulations, revealed that increasing inlet velocity significantly enhances vorticity and fluid flow, raising the mass transfer coefficient by 1.51 to 6.72 times compared to static systems. This approach increased the etching rate of ∼600 nm Si nanoholes by approximately 27.78% and enabled the controllable fabrication of tortuous nanohole arrays [31].

### 2.3. Emerging Fabrication Techniques

#### 2.3.1. Stain Etching (Electroless Chemical Porosification)

Stain etching refers to electroless porous silicon formation driven by solution-phase oxidants (e.g., HNO_3_, Fe^3+^/FeCl_3_, V-containing oxidants) in the presence of HF, where oxidant-mediated hole injection/Si oxidation is coupled to HF-assisted dissolution. In contrast to electrochemical anodization, stain etching does not require an externally applied bias, and the resulting morphology is typically governed by the oxidant/HF ratio, the water content, and the associated transition between reaction-controlled and diffusion-controlled regimes in HF–HNO_3_-based mixtures [32]. In recent years, stain etching has been revisited mainly as a low-complexity route for large-area nanostructuring or for situations where electrical contacting is undesirable, while efforts have focused on improving predictability through composition–kinetics analysis and through hybrid workflows that integrate stain-etched porous silicon into functional stacks.

**Figure 2:**
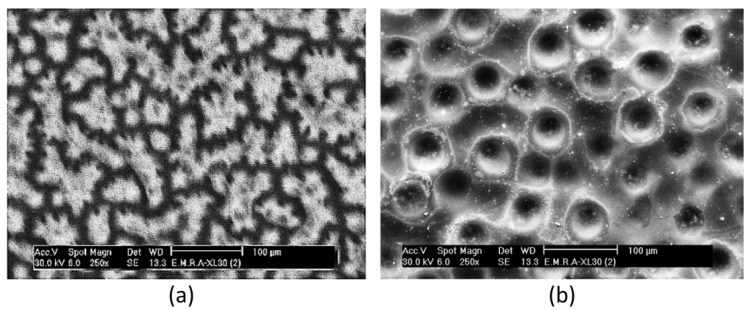
Morphological control in stain etching via catalyst integration. (**a**) Conventional HF/HNO_3_ stain etching results in irregular surface “drillings.” (**b**) Introducing an Ag-assisted process under identical conditions significantly improves pore uniformity, illustrating the sensitivity of morphology to catalytic pathways [33]. Adapted from Mogoda and Farag, Silicon (2022), Figure 2a,b.

A representative HF/HNO_3_ stain-etching window was quantified by Mogoda and Farag, who directly compared stain etching and Ag-assisted chemical etching under identical conditions. In HF/HNO_3_ systems, this morphological sensitivity can be visualized directly through comparative experiments. Mogoda and Farag observed that, under 22 M HF/0.5 M HNO_3_ for 1 h, conventional stain etching produced surfaces characterized by irregular “drillings”; when an Ag-related process was introduced under the same HF/HNO_3_ conditions, the pore morphology and uniformity changed markedly (Figure 2) [33]. Complementary to these morphology-based observations, quantitative studies of the reaction network and rate behavior in HF/HNO_3_ (and HF/HNO_3_/H_2_SiF_6_) mixtures have proposed that variables such as water content can be used to unify etch-rate descriptions and to rationalize composition-dependent differences in both etch kinetics and resulting morphology, thereby providing a framework for moving stain etching from empirical recipes toward more predictable process windows [32].

Recent application-oriented demonstrations frequently employ stain etching as the porous-Si formation step and then decouple functionality through subsequent deposition or modification. For example, Wang et al. explicitly report using a hydrofluoric stain etching method to fabricate nanostructured black silicon, followed by TiN nanoparticle decoration; the resulting PIN photodetector exhibited a responsivity of 0.45 A·W^−1^ at 1060 nm, illustrating how stain-etched nanostructures can be integrated into device stacks without bias-driven porosification [34]. In a chemically functional composite route, a stain etching step was combined with ultrasonication and photoreduction to prepare an Au-decorated porous silicon/poly (3-hexylthiophene) nanocomposite for dopamine sensing; the reported device metrics include a sensitivity of 0.5112 μA·μM^−1^·cm^−2^, a linear range of 1.0–460 μM, and a detection limit of ~0.63 μM, demonstrating stain etching as a scalable porous-Si precursor step in hybrid sensor architectures [35].

Beyond nitrate-based oxidants, Fe^3+^-driven stain etching continues to be explored as a tunable electroless route to luminescent porous silicon. In an AIP Conference Proceedings report (2022), porous silicon formed by stain etching using FeCl_3_ oxidant exhibited photoluminescence features including an intense peak at 234 nm along with visible-region peaks, and SEM-based discussion associated these features with stain-etch-generated nanostructures [36]. A related oxidant engineering example is provided by the Materials Today: Proceedings study on stain etching with V_2_O_5_ and FeCl_3_, where highly doped Si wafers were stain etched in HF containing Fe^3+^/VO_2_^+^ species and visible photoluminescence was observed, with etching time identified as a key variable for optical response evolution [37]. Collectively, these recent examples underscore that contemporary stain-etching development is largely centered on (i) mapping composition/transport sensitivity in HF–oxidant mixtures and (ii) leveraging stain-etched porous Si as a process-simple, bias-free precursor for downstream functionalization [32].

#### 2.3.2. Vapor-Phase Etching

In the context of porous silicon and nanostructured silicon processing, vapor-phase etching generally refers to the selective removal or nanostructuring of silicon under non-liquid immersion conditions, using reactants in the gas or vapor state (typically HF vapor, using air/oxygen as an oxidant or carrier gas). Compared to liquid-phase systems, the technological value of the vapor-phase route is often reflected in two main aspects. Firstly, the supply of reactants and the removal of byproducts can be procedurally modulated via parameters such as flow rate, pressure, temperature, and pulsed injection, thereby reducing the uncertainties associated with the liquid-phase “diffusion boundary layer” during the evolution of high-aspect-ratio structures. Secondly, by avoiding liquid immersion and drying processes, vapor-phase systems offer a distinct advantage in mitigating the risk of structural collapse or stiction caused by capillary forces, making them particularly suitable for preserving high-aspect-ratio array structures and controlling large-area uniformity [38].

In gas-/vapor-phase metal-assisted chemical etching (MacEtch), the principle that the catalyst pattern localizes and defines the reaction front is retained, but the rate-limiting transport pathway shifts from liquid diffusion to a combination of gas-phase mass transfer, surface adsorption/condensation, and interfacial reaction kinetics. In a representative Pt-catalyzed implementation, the etching environment is generated by water-diluted HF vapor together with a continuous air flow (air providing both oxidant and carrier gas), enabling highly directional pattern transfer: Romano et al. reported feature sizes down to 10 nm, straight Si nanowires with 10–100 nm cross-sections and lengths of hundreds of micrometers, and etch depths in the tens-of-micrometers range, with an etch-rate window of ~5–25 μm h^−1^ in their gas-phase process [23].

**Figure 3:**
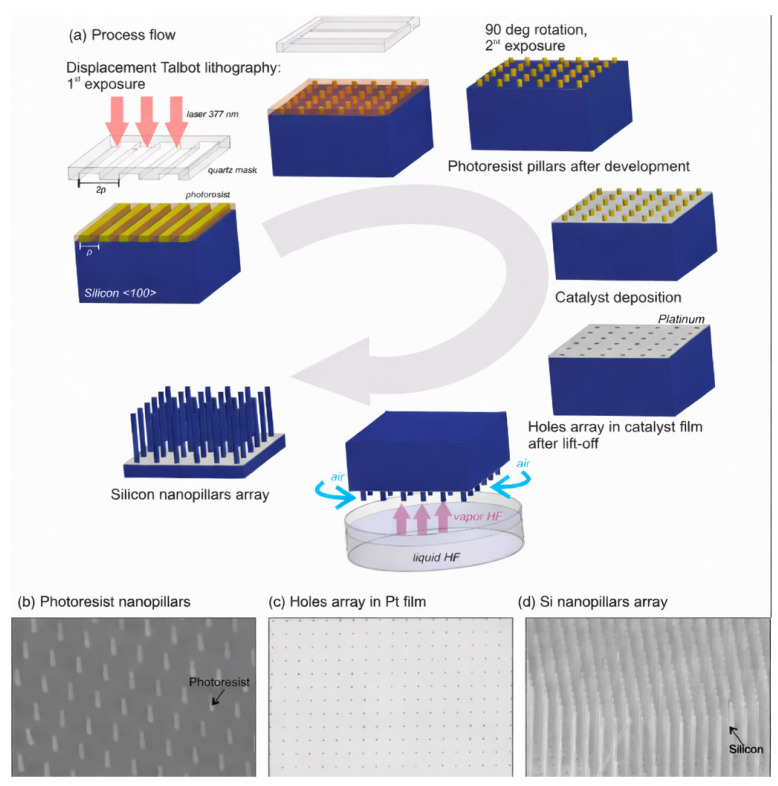
High-aspect-ratio nanopillar fabrication via Displacement Talbot Lithography (DTL) and gas-MacEtch. (**a**) Workflow integrating large-area catalyst patterning with vapor-phase etching. (**b**–**d**) SEM progression showing the precise transfer of the photoresist pattern (**b**) into the Pt catalyst (**c**) and finally into vertical silicon nanopillars (**d**) with aspect ratios > 200 [39]. Scale bars: 1 μm in (**b**,**c**) and 2 μm in (**d**). Reproduced/adapted from Shi et al. (ref. [37]).

Resonating with the aforementioned “single patterned catalyst layer + vapor-phase etching” route is the process integration of coupling vapor-phase MacEtch with scalable nanopatterning methods to achieve “ordered arrays + high aspect ratios.” Shi et al. combined Displacement Talbot Lithography (DTL) with gas-MacEtch to realize ordered nanopillar arrays on high-resistivity Si(100) (1–30 Ω·cm). Their results provide a comprehensive quantitative description: the Pt film hole-array catalyst pattern has a period of approximately 1 μm and hole diameters of 100–250 nm. After vapor-phase etching, Si nanopillars with a scale of about 200 nm were obtained, achieving an aspect ratio > 200, with a reported maximum etch rate of 1 μm·min^−1^ [39]. As shown in Figure 3a, the process chain is explicitly illustrated from DTL exposure (including a second exposure after a 90° rotation) to the formation of photoresist pillar arrays after development, followed by Pt catalyst deposition and lift-off to generate a periodic hole array in the Pt film; the subsequent Gas-MacEtch step is schematically depicted with vapor HF supplied from a liquid HF source beneath the sample and air flowing laterally as the oxidant/carrier. Figure 3b provides a plan-view SEM of the developed photoresist nanopillar array (1 μm scale bar), Figure 3c shows the corresponding periodic hole array in the Pt film (1 μm scale bar), and Figure 3d presents a cross-sectional SEM confirming the vertically transferred Si nanopillar array above the Pt catalyst layer (2 μm scale bar). Together, these panels visually substantiate the linkage between “large-area periodic catalyst patterning” and “vertical pattern transfer by vapor-phase MacEtch,” consistent with the reported geometric parameters and etch-rate performance [39].

Beyond noble-metal catalysts, vapor-phase etching routes have been explicitly leveraged to address microelectronics compatibility constraints in MacEtch, where Au is widely regarded as a detrimental deep-level impurity in Si and thus problematic for CMOS integration. Kim et al. [25] demonstrated that switching TiN-assisted MacEtch from conventional liquid operation to a true vapor-phase (VP) configuration can suppress the non-ideal “inverse-MacEtch” behavior associated with strong TiN–Si interfacial adhesion and instead enable stable forward MacEtch that engraves the TiN mesh pattern into Si, yielding ordered Si nanowire arrays with aspect ratio > 5:1 [25]. Quantitatively, their VP process was mapped across 50–90 °C, and the temperature dependence of the average etch rate followed an Arrhenius trend with an extracted activation energy of 0.78 eV [40]. Regarding “process controllability,” a significant recent trend in vapor-phase MacEtch is advancing gas-phase reaction conditions from “static exposure” to more programmable process control. Janavicius proposed “programmable VP-MacEtch,” emphasizing independent modulation of etchant flow rate, injection/pulse time, and chamber pressure. They further introduced illumination as an extra degree of freedom to achieve photo-enhanced VP-MacEtch, addressing difficulties in stabilizing etch morphology (porosity/bunching) and etch rate uniformity during large-area processing. In review writing, such work is typically categorized as representative of “vapor-phase process engineering”, as its contribution lies not in a single structural parameter, but in elevating vapor-phase etching from material/morphology demonstrations to process window management closer to manufacturing workflows [38].

Beyond deep etching and high-aspect-ratio transfer, vapor-phase routes are also used to construct nanostructured silicon surfaces with optical functions. Taking the vapor-phase silver-assisted chemical etching (VP-Ag-ACE) reported by Amri et al., the authors explicitly state that this method is suitable for constructing nanostructures on highly doped p-Si and correlating structure with optical properties. Both the abstract and section introduction provide key performance statements: the vapor-etched layer can exhibit reflectivity below 3% in the visible range and display strong multi-peak visible photoluminescence (PL) [41]. These results suggest that vapor-phase systems serve not only “deep structural etching to avoid collapse” but can also act as structural preparation routes for nanostructured silicon surfaces in functional directions such as anti-reflection and light emission [41].

Further addressing the needs for higher resolution and complex pattern transfer in the gas/vapor phase, recent process modifications have increasingly focused on how the catalyst itself is patterned and stabilized prior to etching. For example, an entirely CMOS-compatible ruthenium (Ru) catalyst processing route has been reported, emphasizing dry-etch-based catalyst patterning (rather than lift-off) and showing MacEtch pattern transfer with feature dimensions down to 50 nm, while also demonstrating that controlled oxidation of Ru can moderate catalytic activity and improve uniformity over larger patterned areas [42].

#### 2.3.3. Nanosphere Lithography

As a low-cost nanopatterning route centered on colloidal microsphere self-assembly, Nanosphere Lithography (NSL) typically assumes the role of “large-area periodic pattern definition” in the fabrication of porous silicon and silicon nanostructures. When coupled with Metal-Assisted Chemical Etching (MACE/MacEtch), the metal nanoholes or nanomeshes defined by the microsphere array serve as masks that are further transferred along the normal direction into high-aspect-ratio nanowires, nanoholes, or nanopillar arrays. Compared to high-end lithography techniques such as electron beam or deep ultraviolet lithography, the advantages of NSL are primarily manifested in (i) the ability to obtain periodic array patterns on the centimeter-to-wafer scale, (ii) the continuous tunability of feature sizes via microsphere diameter selection and post-processing (e.g., plasma shrinking, vapor-phase corrosion shrinking), and (iii) a relatively low threshold for process coupling with wet etching. However, it must be emphasized that the “spherical symmetry of the template particles” tends to strongly couple the cross-sectional geometry to a near-circular shape. Furthermore, the grain boundaries and defect density inherent in large-area monolayers directly feed back into the continuity of the metal film and the uniformity of etching. Therefore, the crux of the NSL–MACE system lies not in “whether it can etch,” but in “whether predictable array transfer can be achieved under the constraints of defects, mass transport, and catalytic film stability”.

**Figure 4:**
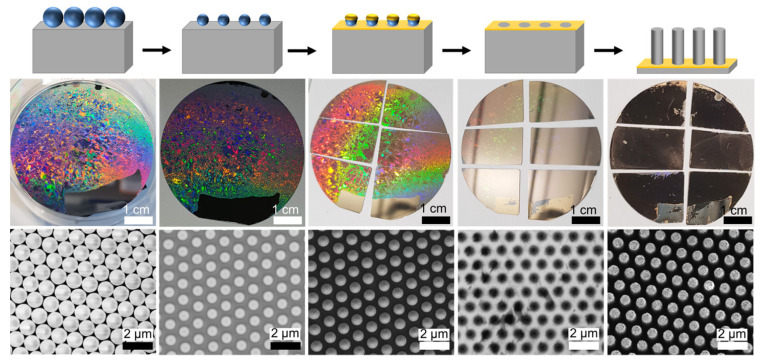
Wafer-scale fabrication of Si nanowire arrays using Nanosphere Lithography (NSL). The schematic (**Top**) and photographs (**Middle**) illustrate the process evolution from a self-assembled polystyrene monolayer to a macroscopic “black silicon” surface. (**Bottom**) SEM images reveal the high microscopic uniformity of the nanowires, confirming that defect-free catalyst meshes are critical for scalable etching [43].

A representative quantitative case study at the wafer scale is provided by Wendisch et al., who applied a polystyrene (PS) microsphere monolayer for the patterning of 2-inch Si wafers. Figure 4 presents the comprehensive fabrication workflow, explicitly correlating the schematic mechanism with macroscopic wafer-scale appearance and microscopic SEM morphology. The process initiates with the self-assembly of a hexagonally close-packed monolayer of PS spheres (d = 1100 nm), which displays distinct opalescence arising from Bragg diffraction (Figure 4, column 1). Subsequent oxygen plasma treatment reduces the sphere diameter to 780 nm to define the mask spacing (column 2), followed by Au deposition (column 3) and template removal to expose a periodic antidot mesh (column 4). In the final stage, MACE transforms the planar pattern into vertical Si nanowire arrays, a change macroscopically marked by the transition from a metallic gold surface to a matte “black silicon” appearance (column 5). Based on this visualizable workflow, the study established a clear “uniformity–stability” process window: under specific conditions, when the vertical etch rate was below approximately 0.6 μm·min^−1^, more than 85% of the samples maintained a “flat, continuous metal film” and exhibited higher macroscopic uniformity. Conversely, at higher rates (>1 μm·min^−1^), the metal film was more prone to bending or fracturing, leading to large-area non-uniformity. Their quantitative comparison also revealed that between arrays with 590 nm and 1500 nm pitches, the etch rate of the former could be higher by about 0.2–0.3 μm·min^−1^, which was attributed to differences in mass transport caused by variations in the Au/Si/enchant three-phase interface length. In addition, the experiment covered wire diameters ranging from 120 to 1050 nm and explicitly pointed out that increasing the adhesion layer thickness reduced both nanowire length and etch rate. More importantly, the article elevated “colloidal monolayer grain defects” to a quantifiable source of failure: when the grain size is small (<100 μm^2^), the elevated density of grain boundary defects significantly weakens metal film stability and degrades the reliability of pattern transfer, providing direct engineering criteria for NSL to move from “feasible” to “scalable [43]”.

Regarding “geometric designability,” the traditional limitation of NSL (spherical template → circular hole/pillar) has seen operational decoupling strategies in recent years. Rey et al. leveraged the compressible self-assembly characteristics of SiO_2_–PNIPAM core–shell particles at the air–liquid interface to achieve relatively independent control over particle size and spacing. After transferring the monolayer at a surface pressure of 7 mN·m^−1^, a non-close-packed array with an inter-particle spacing of approximately 1140 nm was obtained; subsequently, “shadow effect” nanoholes were constructed via glancing angle evaporation, thereby continuously tuning the metal holes from circular to elliptical. The paper provides clear quantitative geometry; for example, under 65° evaporation conditions, the major/minor axes of the elliptical holes reached 336 ± 14 nm/133 ± 10 nm (an axis ratio of approximately 2.6), whereas at 0° evaporation, the holes remained approximately circular (about 159 ± 8 nm). The critical significance of this strategy lies in breaking the strong “spherical template–circular cross-section” coupling through deposition geometry alone, without changing the microsphere material system, and further obtaining Si nanowire arrays with elliptical or crescent cross-sections (while introducing controllable anisotropic collapse/bundling morphologies) during subsequent MACE [44].

Beyond designability at the “single nanowire cross-section” level, NSL–MACE is also being used to construct higher-order array primitives, thereby serving hotspot structures required for applications such as sensing and optical field enhancement. Bartschmid et al. combined colloidal lithography + MACE with directed nanoparticle assembly to construct monomer/dimer/tetramer arrays of vertical Si nanowires, and assembled a monolayer of Au nanoparticles on the array surface for SERS. The structural scale provided in the abstract has direct “manufacturability” implications: Au nanoparticles of approximately 20 nm were able to enter the ~40 nm gaps between Si nanowire oligomers, thereby effectively reaching electromagnetic hotspot regions; this allowed for a comparison of the Raman response differences among various oligomer primitives under 785 nm excitation (reporting a “dimer best” structure–property correlation). In the context of a review, the value of such results is to demonstrate that NSL is not necessarily limited to “hexagonally periodic single-pillar arrays,” but can synergize with post-assembly/secondary patterning to advance arrays from “regular periodic structures” to “programmable primitive libraries [45]”.

It is worth noting that the “continuous size tunability” of NSL does not rely solely on plasma shrinking; in specific workflows coupled with MACE, practices utilizing HF vapor to reduce the size of SiO_2_ microspheres—a gentler method for refining feature sizes—have also emerged. Taking Nguyen et al. as an example, they employed 235 nm SiO_2_ nanospheres to form a hexagonally close-packed monolayer via drop-casting on a tilted substrate, reduced the microsphere size via infrared-assisted HF vapor etching, and subsequently coupled this with MACE to prepare ordered Si nanopillar arrays. The work emphasized that “pillar height is controlled by etching time, while diameter is defined by NSL,” and systematically characterized their optical responses, including reflectance, room-temperature PL, and Raman enhancement. The methodological significance of this type of workflow is that when the target moves from “sub-micron pillars” to even smaller linewidths, achieving secondary template scaling via a vapor-phase step—without introducing significant risks of liquid-phase capillary collapse—can serve as an effective size-tuning knob in the NSL–MACE process chain [46].

Finally, addressing the demand for “hierarchical and functionally encoded structures,” the evolution of NSL–MACE extends beyond lateral template design to the dynamic control of the vertical etching profile. Kismann et al. demonstrated a strategy to tailor the longitudinal geometry of Si nanostructures by temporally modulating the HF/H_2_O_2_ concentration ratio, without the need to modify the initial noble metal catalyst. This approach enables the distinct formation of “Si nanopencils” and nanopillars with single or multiple constrictions, achieving high aspect ratios (up to 27) with relative diameter constrictions of approximately 0.6. The functional implications of such geometric engineering are quantifiable: the multiple constrictions significantly suppress thermal conductivity via phonon backscattering, providing a direct pathway for advanced thermoelectric applications, while the pencil-like morphologies yield superior broadband antireflection properties (reflectivity < 3% in visible–IR ranges). This work underscores a critical methodological shift; by integrating NSL with dynamic wet-chemical parameters, the process moves from generating simple isotropic cylinders to manufacturing complex, diameter-modulated 3D architectures optimized for specific thermal or photonic transport management [47].

In summary, the “emerging nature” of NSL in this field is not embodied in the novelty of its principle, but rather in its gradual formation of a scalable process chain of “large-area periodic pattern definition → catalytic film stability/mass transport window control → high-fidelity 3D array transfer” following its coupling with wet (or vapor) etching techniques such as MACE. Key progress in recent years has focused on two points: first, wafer-scale uniformity and failure modes (grain defects, metal film fracture, mass-transport-induced rate thresholds) have been quantified and translated into actionable window criteria; second, through strategies such as tilted deposition, core–shell particle assembly, and post-assembly, the strong geometric coupling caused by spherical templates has been partially broken.

## 3. Surface Chemistry and Functionalization

### 3.1. Surface Instability of As-Prepared Hydride Surfaces

Electrochemically etched porous silicon (PSi) typically possesses a hydride-terminated surface (Si–Hx). The chemical reactivity of this interface is largely dictated by the thermodynamic instability of surface Si–H and Si–Si bonds against oxidation. This thermodynamic drive facilitates a spontaneous evolution toward oxide species (Si–OH/Si–O–Si) in the presence of air, moisture, or chemical oxidants [48]. In the context of the high specific surface area and high density of surface defects characteristic of the porous scaffold, this “spontaneous oxidation” is significantly amplified. Consequently, the temporal stability of the “as-prepared” Si–H surface constitutes a fundamental challenge that must be prioritized in applications ranging from sensing and energy storage to microelectronics [48].

From the perspective of air and humidity-induced oxidation, recent quantitative characterizations of hydrogen-terminated silicon nanocrystals provide direct evidence of signal drift. Ghosh and Shirahata monitored silicon nanocrystals (~2 nm diameter) and observed that the infrared intensity ratio of Si–H/Si–O decreased from 0.60 to 0.40 after 48 h of air exposure. Concurrently, the photoluminescence (PL) peak exhibited a blue shift (821 nm to 811 nm) alongside a reduction in quantum yield (10.6% to 7.6%), indicating that surface oxidation alters optical response even under relatively mild environmental conditions [49]. Similarly, Kolasinski et al. highlighted that the significant oxidation/hydrolysis of PSi in humid air or aqueous environments directly correlates with the sensitivity of PL signals to rinsing and thermal history, providing a material-level basis for the “exposure-evolution-drift” chain [50].

In contrast to the relatively slow aging observed in ambient air, chemical oxidants trigger rapid surface transformations resolvable on the minute scale. León-Valiente et al. (2025) reported that upon H_2_O_2_ treatment, the PL component associated with Si–Hx species decayed from ~80% to ~35% within just 20 min, while the oxide-related component surged from ~16% to ~57% [51]. Kinetic analysis revealed a first-order decay constant for Si–H (k = 0.0938 min^−1^) that was markedly higher than the formation rate of Si–O–Si, underscoring the intrinsic lability of the hydride surface under oxidative stress [51]. This dynamic instability reflects broad temporal evolution patterns [52], necessitating rigorous surface engineering comparable to quantum dot tuning [53].

**Figure 5:**
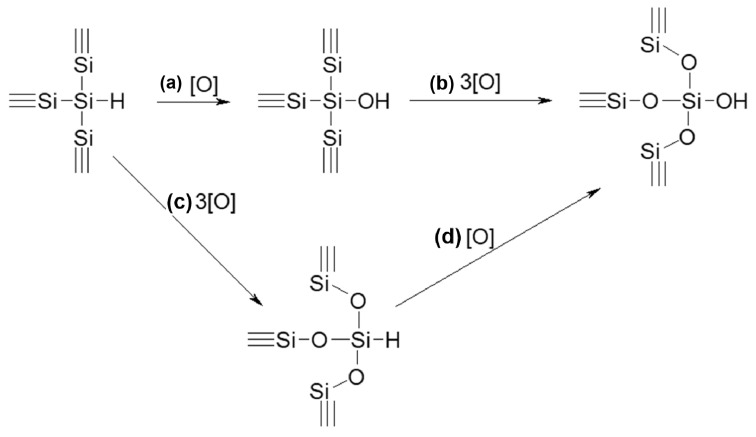
Oxidation pathways of hydride-terminated silicon in aqueous media. The scheme illustrates how surface hydrolysis (path a) and back-bond oxidation (path c) competitively consume the silicon skeleton, leading to hydrogen generation and the formation of a silica-like network [54].

This surface instability is further manifested in aqueous media, where it often couples with hydrogen evolution and structural oxidation. Mussabek et al. systematically investigated the oxidation mechanism of porous silicon nanopowders in water. As illustrated in Figure 5, the oxidation of the hydride-terminated surface (≡Si–Si–H) proceeds via competitive pathways: direct hydrolysis of the surface Si–H bond (path a) or oxygen insertion into the Si–Si back-bonds (path c), both ultimately converging toward a silica-like structure. The authors noted that the high surface hydrogen content (6 wt%) can enhance the volume of H_2_ generated via these reactions by approximately 1.5 times, a process active even at sub-zero temperatures [54].

Furthermore, Ning et al. showed that metal-assisted chemically etched (MACE) Si nanowire arrays generate hydrogen in water at room temperature—without external energy input—at rates nearly an order of magnitude higher than other reported nanostructures [55]. These findings corroborate recent reviews suggesting that long-term stability in aqueous/alcoholic solutions cannot be assumed for hydride-terminated silicon; rather, it necessitates predictable interface reconstruction via passivation or controlled oxidation [56].

It is important to emphasize, however, that oxidation is not exclusively deleterious; “controlled oxidation” is often employed to convert the labile Si–H surface into a robust oxide layer for specific applications. For instance, Castillo Calvente et al. (2025) utilized a two-step thermal (400–600 °C) and aqueous oxidation protocol to stabilize electrochemically etched PSi particles [57]. By monitoring luminescence over 35 days, they demonstrated that such treatments are essential for achieving sustained optical performance and biocompatibility. The very necessity of this secondary surface transformation serves as indirect yet compelling evidence of the inherent instability of the pristine Si–H surface [57].

In summary, the oxidation of hydride-terminated PSi represents a multi-scale instability issue coupling chemical bonds, pore walls, and device signals. Whether manifesting as significant drifts in composition and luminescence over 24–48 h in air, rapid kinetic decay in chemical oxidants, or hydrogen evolution in water, this “propensity for in situ oxidation” must be explicitly defined as a boundary condition. Consequently, surface passivation or functionalization strategies are indispensable for ensuring reproducible sensing responses and long-term device reliability.

### 3.2. Stabilization Strategies

In response to the intrinsic instability of as-prepared hydride-terminated surfaces (Si–Hx) discussed in Section 3.1—specifically their propensity for spontaneous oxidation and hydrolysis in ambient environments—robust surface engineering is prerequisite for practical application. The predominant stabilization strategies reported in the literature can be broadly categorized into three distinct pathways: (i) controlled thermal oxidation, which replaces metastable hydrides with a thermodynamically stable silicate network (Si–O–Si); (ii) organic functionalization via hydrosilylation, which establishes kinetically robust Si–C bonds; and (iii) conformal coating techniques, including carbonization and Atomic Layer Deposition (ALD). Fundamentally, the objective of these protocols extends beyond the mere suppression of oxidation. Rather, they aim to convert the unpredictable, spontaneous surface evolution into a chemically programmable interface, thereby securing the reproducible baselines and operational longevity required for high-fidelity sensing, energy storage, and microelectronic integration.

#### 3.2.1. Controlled Thermal Oxidation: From Si–H Instability to Programmable Si–O–Si Interfaces

Among the strategies for stabilizing porous silicon, controlled thermal oxidation represents the most systematic and process-predictable pathway. Its fundamental value extends beyond the mere formation of a physical oxide capping layer; rather, it relies on the thermodynamic conversion of the reactive, hydride/back-bond dominated surface into a robust siloxane (Si–O–Si) network through the precise regulation of oxidation temperature and duration. As noted by Sailor, this chemically driven interface reconstruction serves as a critical bridge advancing PSi from a “lab-scale material” to an “engineering platform,” as it establishes the thermodynamic foundation for a repeatable operational baseline [48].

**Figure 6:**
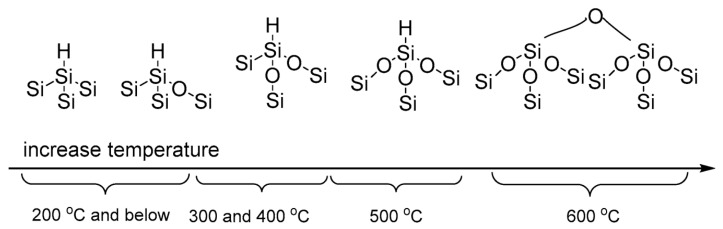
Temperature-dependent surface evolution during thermal oxidation. The schematic depicts the transition from a labile hydride surface (<200 °C) to a stable, fully cross-linked siloxane network (Si–O–Si) at 600 °C, a transformation that directly correlates with improved hydrolytic stability [58].

From the perspective of stability, the most direct impact of thermal oxidation is observed in the modulation of hydrolytic kinetics. Wang et al. utilized mesoporous silicon particles in physiological buffers (pH 7.2) to demonstrate that while unoxidized PSi undergoes rapid dissolution within minutes, samples oxidized at temperatures 300 °C exhibit a significantly retarded, quasi-linear mass loss. As illustrated in Figure 6, this kinetic transition is structurally underpinned by the evolution of surface chemistry: below 200 °C, the surface retains a high population of metastable Si–H bonds; as temperatures reach the 300–400 °C range, oxygen insertion into the back-bonds becomes prevalent, stabilizing the silicon skeleton; finally, at temperatures approaching 600 °C, the surface is dominated by a condensed siloxane network Si–O–Si. Quantitative analysis by Wang et al. confirmed that the “fast dissolution component” associated with residual Si–H species is effectively suppressed at these higher oxidation temperatures, reducing the dissolution rate constant by up to three orders of magnitude. These findings establish the degree of oxidation as a first-order parameter that defines the “lifetime window” of PSi in aqueous environments, rather than serving merely as a post-processing step [58].

However, this enhancement in physical stability is invariably accompanied by a fundamental reshaping of the surface electronic structure and transduction mechanism. In a study on isopropanol vapor sensing, Baran et al. revealed that increasing the oxidation temperature (423–723 K) not only alters the magnitude of the resistive response but also inverts its polarity, transitioning from a weak positive response characteristic of the hydride surface to a negative response typical of metal oxide semiconductors. The authors attributed this inversion to shifts in carrier transport mechanisms and surface adsorption sites. This underscores a critical implication for sensor design: thermal oxidation is not a “neutral” stabilization method; rather, it actively dictates the signal generation mechanism itself [59].

In the realm of photonic structures, thermal oxidation functions as a precision knob for tuning refractive index and optical loss. Ramírez-Gutiérrez et al. demonstrated that by carefully controlling oxidation conditions (600–1000 °C) in one-dimensional photonic crystals, the gradual conversion of pore walls to silica can be leveraged to minimize absorption losses while preserving the designed photonic bandgap [60]. Pushing this strategy to its limit, Lujan-Cabrera et al. showed that PSi can be fully converted into porous quartz (PQz) while retaining its original geometric morphology. This “terminal oxidation state” exhibits minimal spectral drift at high temperatures, effectively offering a strategy of “chemical replacement with geometric retention” for applications in harsh environments [61].

Consequently, in the construction of integrated devices, thermal oxidation is often embedded as a requisite “surface-state reset” strategy. For instance, Chen et al. employed rapid thermal oxidation (800 °C) as a pretreatment for ZnO/PSi biosensors, enhancing both aqueous stability and fluorescence reproducibility [62]. This pursuit of environmental durability shares key stability principles with modified zeolites [63] and composite nanofibers [64]. Collectively, these findings suggest that thermal oxidation systematically redefines the material’s dissolution kinetics, carrier transport, and optical baselines via the controlled Si–H to Si–O–Si transformation. The selection of “optimal” oxidation conditions is therefore highly application-dependent, prioritizing kinetic inhibition for liquid sensing, balancing sensitivity versus stability for gas sensing, or serving as a material conversion tool for photonics. In this sense, thermal oxidation constitutes the chemical baseline against which more complex strategies, such as hydrosilylation and atomic layer deposition, are evaluated.

#### 3.2.2. Hydrosilylation: Constructing Hydrolytically Robust Si–C Monolayers

In contrast to thermal oxidation, which “resets” the surface state through the formation of an inorganic Si–O–Si network, hydrosilylation represents a fundamentally different stabilization pathway. It directly exploits the reactive Si–H bonds on the as-prepared PSi surface as active sites, inserting unsaturated molecules (alkenes or alkynes) to form Si–C covalent bonds that are significantly more hydrolytically inert. The essence of this process lies in transforming a “hydride interface susceptible to continuous erosion by water and oxygen” into a “passivated interface dominated by an organic monolayer.” As summarized in recent reviews, the Si–C bond is widely regarded as one of the most stable chemical linkages available for silicon surfaces, making hydrosilylation the primary route for achieving high-coverage monolayer passivation. Concurrently, it is established that the material’s form factor—whether thin films, particles, or hierarchical pore structures—significantly modulates the reaction window and the upper limit of surface coverage [65].

From the perspective of stability requirements, biologically relevant environments provide the most direct stress test scenarios. Under physiological ionic strength and near-neutral pH, the Si–H/Si–Si back-bonds of unmodified PSi are prone to continuous oxidation and hydrolysis, leading to irreversible drifts in optical or electrical baselines. Consequently, hydrosilylation is often positioned at the very front of the “stabilization–refunctionalization” process chain to establish a reproducible chemical foundation. A representative example involves the mild hydrosilylation of PSi nanoparticles (PSiNPs) with undecylenic acid. Studies have verified that this covalent coupling significantly preserves luminescence and structural integrity under simulated physiological conditions (PBS, 0.1 M, pH 7.4). Such designs not only provide necessary passivation via Si–C anchoring but also reserve terminal carboxyl groups for subsequent bioconjugation, thereby achieving a dual benefit of stability and bioactivity [66].

**Figure 7:**
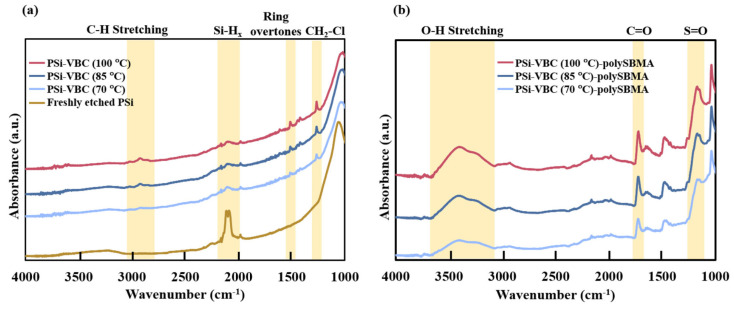
Hierarchical surface modification verified by ATR-FTIR. (**a**) Thermal hydrosilylation of vinylbenzyl chloride (VBC) suppresses Si–Hx bands (~2100 cm^−1^) and introduces Si–C bonds. (**b**) Subsequent grafting of polySBMA is confirmed by characteristic amide and sulfonate bands, demonstrating the construction of a robust antifouling interface [67]. Reprinted with permission from Smail et al., ACS Omega 2025, 10, 31932–31939.

When the application objective shifts from particle survival to sustained device readout, hydrosilylation becomes a key strategy for suppressing baseline drift and enabling scalable interfacial engineering. Smail et al. reported a representative protocol in which a stable organic interlayer is first formed on PSi via thermal hydrosilylation of vinylbenzyl chloride (VBC), generating a covalent Si–C anchor that subsequently serves as an initiation platform for grafting a zwitterionic antifouling polymer (polySBMA). The stepwise construction of this interface is directly evidenced by ATR-FTIR spectroscopy (Figure 7). As shown in Figure 7a, hydrosilylation leads to attenuation of the Si–Hx stretching band near ~2100 cm^−1^, accompanied by the emergence of C–H stretching modes and aromatic ring overtones, confirming Si–C bond formation. After polymer grafting, Figure 7b displays characteristic O–H, C=O, and S=O vibrational bands associated with polySBMA, verifying successful formation of the secondary functional layer. This “Si–C anchor layer → functional polymer layer” architecture was shown to markedly reduce non-specific adsorption and stabilize the sensing baseline in complex media, highlighting hydrosilylation as a critical enabling step for converting PSi into an engineerable grafting platform for reliable biosensing readout [67].

The stabilization benefits conferred by hydrosilylation are not confined to mild aqueous conditions; in highly reactive or aging-prone systems, its advantages are often manifested in quantifiable functional retention. Addressing the performance degradation of nanoporous silicon (nPS) caused by slow oxidation in air, the Chemical Engineering Journal reported a strategy utilizing thermal hydrosilylation to construct dense alkyl self-assembled monolayers (SAMs). Using the exothermic enthalpy as a direct stability metric for high-porosity (~79.6%) nPS films, the study showed that modified samples retained their heat of reaction (~1402–1387 J·g^−1^) with almost no significant decay after 24 to 48 h of accelerated aging, compared to the initial value (~1436 J·g^−1^). This direct mapping from “microscopic surface chemical stability” to “macroscopic functional output” provides compelling evidence for the engineering value of Si–C passivation [68].

While traditional protocols often necessitate high temperatures, UV irradiation, or chemical initiators, a significant recent trend involves expanding hydrosilylation into a more versatile and milder “reaction toolbox.” For instance, Benjamin et al. reported a strategy for the room-temperature alkylation of silicon nanocrystals (e.g., with dodecene), highlighting a distinct size-dependence in reaction kinetics which suggests that nanoscale curvature effects or surface radical chemistry can be leveraged to lower activation barriers [69]. Furthermore, Nanoscale Advances proposed an ultrasound-assisted room-temperature hydrosilylation scheme: under standard ultrasonic bath conditions, a surface coverage approaching 30% can be achieved within 24 h, and reaction efficiency can be quadrupled with the addition of trace radical initiators. These advances indicate that hydrosilylation is evolving from a single-route process dependent on harsh conditions into a scalable, easily integratable surface treatment triggerable by physical fields or trace initiators [70].

Finally, hydrosilylation is frequently employed to directly “write” specific interfacial wettability or anti-fouling attributes, which reciprocally enhances structural and signal stability. For example, research utilizing the photocatalytic hydrosilylation of fluorinated methacrylates (e.g., dodecafluoroheptyl methacrylate) on n-type PSi has yielded modified materials combining high stability with superhydrophobic characteristics. The design logic here extends beyond physical coverage by an organic layer; it exploits low-surface-energy termini to significantly reduce the wetting depth and reaction probability of aqueous media, thereby mitigating structural collapse and signal drift via a physical mechanism. Such work reinforces the concept that hydrosilylation provides not merely chemical bond stability, but also programmable interfacial physical properties (wetting, anti-fouling), effectively coupling stabilization and functionalization within a single molecular layer design [71].

#### 3.2.3. Carbonization and Atomic Layer Deposition: From Interfacial Transformation to Conformal Encapsulation

When porous silicon (PSi) devices are required to operate under sustained aqueous, electrochemical, or bias-driven conditions, surface passivation strategies based solely on Si–O–Si or Si–C termination often prove insufficient due to localized defect amplification and pore-to-pore heterogeneity. To address these limitations, stabilization approaches have shifted from simple bond-level passivation to structure-level isolation, achieved either through near-surface material transformation or conformal encapsulation. Thermal carbonization converts the near-surface region of PSi into a silicon–carbon–rich interface, effectively eliminating reactive Si–H and Si–Si back-bonds. Salonen and Mäkilä emphasized that thermally carbonized porous silicon (TCPSi) exhibits significantly superior chemical stability compared with hydrogen-terminated or oxidized PSi, enabling reproducible optical and electrical behavior over extended exposure to air and aqueous media [72].

Beyond stability as an intrinsic material property, carbonization directly supports sustained device readout. Layouni et al. demonstrated that TCPSi-based optical DNA sensors retained signal fidelity under repeated aqueous measurements, whereas oxidized PSi suffered progressive baseline drift [73]. Similarly, in electrochemical contexts, Pérez-Ràfols et al. reported that carbon-stabilized PSi electrodes exhibited stable voltammetric responses with detection limits below 5 μM and high recovery rates in tap-water samples, highlighting the effective suppression of corrosion-induced signal degradation [74]. Furthermore, Pei Chin et al. utilized thermally hydrocarbonized PSi for the label-free electrochemical detection of bacterial RNA in serum-containing media, confirming the suitability of carbonized interfaces for complex biological environments [75]. Collectively, these results indicate that carbonization redefines the chemical identity of the pore walls, thereby decoupling long-term device performance from the intrinsic reactivity of the silicon skeleton.

**Figure 8:**
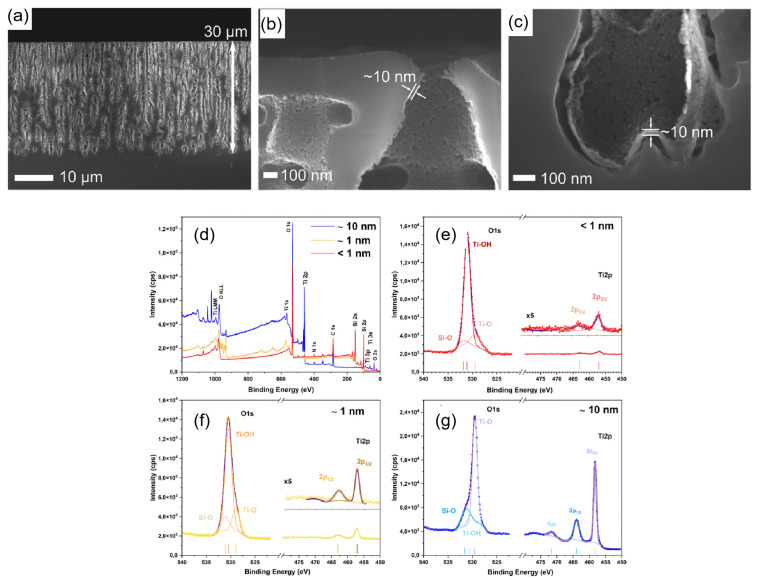
Conformal passivation of high-aspect-ratio pores via Atomic Layer Deposition (ALD). (**a**–**c**) Cross-sectional SEM images demonstrating uniform TiO_2_ coating (~10 nm) throughout 30 μm deep macropores. (**d**–**g**) XPS analysis tracking the precise stoichiometric evolution from sub-monolayer coverage to continuous films, verifying ALD’s capability to overcome diffusion limits in deep channels [76].

In contrast to the material transformation of carbonization, atomic layer deposition (ALD) provides an orthogonal stabilization strategy by depositing ultrathin, conformal oxide layers throughout high-aspect-ratio pore networks. This approach preserves the silicon framework while introducing a controllable diffusion barrier independent of pore geometry. The precision of this approach is exemplified by Al Chimali et al. [76]. (Figure 8), where cross-sectional SEM analysis confirms that a uniform ~10 nm TiO_2_ coating is maintained throughout ~30 μm deep macropores (Figure 8a–c), effectively overcoming mass transport limitations in high-aspect-ratio geometries. Complementary XPS analysis (Figure 8d–g) quantifies this process, validating the precise stoichiometric evolution from sub-monolayer coverage (<1 nm) to continuous films (~10 nm). Building on this structural control, Vansweevelt et al. demonstrated that ALD passivation (using Al_2_O_3_, TiO_2_, and HfO_2_) significantly reduced optical noise, enabling rapid detection of Bacillus cereus lysates [77]. Functionally, Al Chimali et al. further showed that even sub-nanometer coatings (<5 cycles) were sufficient to suppress photo-corrosion, while thicker layers (~10 nm) enhanced photocatalytic activity without sacrificing pore accessibility. Collectively, carbonization and ALD represent complementary endpoint strategies—providing maximal interfacial robustness and unparalleled conformality, respectively—that are increasingly employed to decouple long-term device performance from the intrinsic reactivity of the silicon skeleton [76]. Fundamental studies have further confirmed that ALD enables uniform coating of both macro- and mesoporous silicon with precise thickness control, providing a reproducible foundation for integration into microelectronic and photonic architectures.

From a design perspective, carbonization and ALD represent complementary endpoint strategies for demanding environments: carbonization offers maximal chemical robustness through interfacial transformation, while ALD provides unparalleled conformality via external encapsulation. Current research trends increasingly combine these approaches with upstream surface treatments to optimize nucleation and interface quality, reflecting a broader shift toward hierarchically engineered PSi interfaces for reliable long-term operation.

### 3.3. Bio-Interface Engineering: Modification for Selectivity in Biosensing

While fundamental stabilization strategies like carbonization and atomic layer deposition (ALD) provide the necessary chemical robustness, the functionalization of porous silicon (PSi) for specific bio-applications requires a higher level of surface engineering. Just as the field of organic synthesis has evolved toward complex axially chiral ligands for asymmetric catalysis [78], PSi surface chemistry is moving from simple alkyl monolayers to structurally precise, multifunctional interfaces. As PSi evolves from a material viable in simple buffers to a device capable of reproducible readout in complex biological matrices, the scope of surface chemistry expands from maximizing binding capacity to establishing signal discrimination at the device level. This transition necessitates a dual strategy within the surface functionalization paradigm, suppressing background noise via advanced antifouling chemistries and optimizing the physicochemical environment of recognition elements to ensure that target occupancy generates a distinct signal.

A critical challenge in surface functionalization is defining selectivity within the operational window of complex fluids. Awawdeh et al. addressed this by comparing polyethylene glycol (PEG) with zwitterionic peptides as antifouling coatings for aptamer-based lactoferrin detection. The zwitterionic interface proved superior, achieving a signal-to-noise ratio (SNR) of ~40 in gastrointestinal fluid compared to negligible SNRs for non-target proteins, thereby demonstrating that intrinsic antifouling chemistry is essential for suppressing interference-induced optical noise. Building on this chemical foundation, the authors further demonstrated that surface chemistry must be coupled with mass transport engineering. By integrating the sensor into a microfluidic architecture with active mixing, they achieved a limit of detection (LOD) of 50 nM—over an order of magnitude improvement. This underscores that in porous media, the “functional surface” is a dynamic system where intrinsic antifouling chemistry works in concert with boundary layer regulation to minimize false signals [79]. Complementing this optical approach, Pei Chin et al. utilized morphological engineering to enhance electrochemical sensitivity. By applying an insulating Si_3_N_4_ coating to thermally carbonized PSi and optimizing pore dimensions, they confined current to functionalized pores. This architecture maximized the signal from pore blockage, achieving a 2.3 pM LOD for bacterial RNA in serum [75].

Beyond regulating biological interactions, surface chemistry is increasingly employed to construct synthetic recognition sites directly on the pore walls. Molecular Imprinted Polymers (MIPs) offer a robust alternative to labile biomolecules, essentially “outsourcing” selectivity to a stable polymeric network. Nocerino et al. utilized electropolymerization to deposit a ~5 nm MIP layer on PSi for the label-free detection of IL-6. This approach relies on the specific coupling between the polymer’s imprinted cavities and intra-pore diffusion, achieving an LOD of 13 nM in bovine serum. This represents a shift in functionalization strategy, where the “recognition element” is no longer an immobilized molecule but an integral part of the surface coating itself [80]. Complementing synthetic strategies, Yaghoubi et al. utilized cost-effective lectins as antibody alternatives for pathogen discrimination. By functionalizing meso-PSiO_2_ with specific lectins (ConA, WGA), they established a RIFTS platform that exploits differential glycan recognition—specifically, ConA targeting Gram-negative Escherichia coli and WGA targeting Gram-positive Staphylococcus aureus—to achieve a limit of detection (LOD) of ~10^3^ cells mL^−1^. Coupled with Principal Component Analysis (PCA), this work validates the efficacy of this antibody-free strategy for bacterial Gram-typing [81].

**Figure 9:**
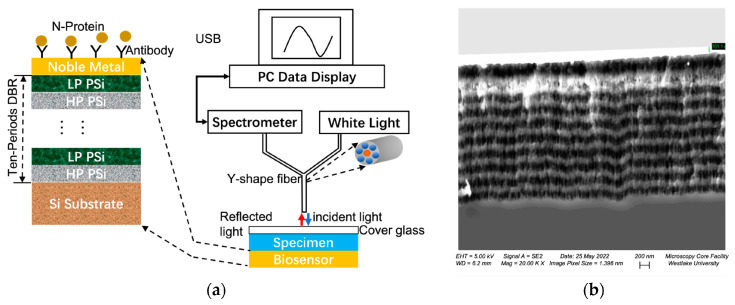
Opto-chemical sensor design based on Tamm Plasmon Polaritons (TPP). (**a**) The architecture combines a porous silicon Bragg reflector with a functionalized noble metal cap to support TPP modes. (**b**) Cross-sectional SEM confirms the periodic stratification required for high-Q optical confinement and sensitive N-protein detection [83].

Functionalization strategies are also evolving to include biochemical pre-processing and optical field coupling. Vercauteren et al. proposed a “lysis-then-readout” strategy, functionalizing the interface not for capture, but for downstream detection of lysates generated by phage endolysins. This effectively redefines the surface’s role from “catching whole bacteria” to detecting controllable molecular fragments, thereby circumventing pore blockage [82]. Parallel to this, Guo and Sun demonstrated the synergy between opto-chemical interface integration, utilizing a Tamm plasmon polariton (TPP) structure as illustrated in Figure 9. As shown in the device schematic (Figure 9a), the sensor consists of a periodic porous silicon distributed Bragg reflector (DBR) capped with a noble metal layer to support the TPP mode. The structural fidelity of this optical cavity is confirmed by the cross-sectional SEM image (Figure 9b), which clearly reveals the alternating high- and low-porosity layers essential for high-Q confinement. By utilizing Protein A to orient antibodies on the metal surface, steric hindrance was reduced, while the strong local field of the TPP mode amplified the specific binding signal. This coupled design achieved >90% sensitivity and >95% specificity for the detection of SARS-CoV-2 N-protein [83].

## 4. Advances in Sensing Applications

### 4.1. Optical Sensing Mechanisms

**Figure 10:**
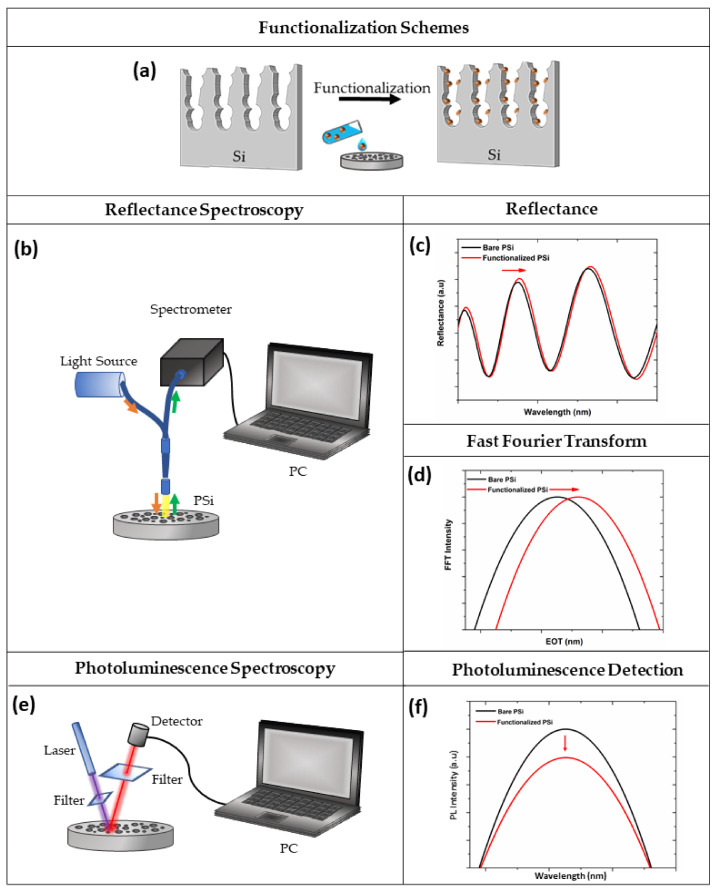
Fundamental optical transduction mechanisms in porous silicon. (**a**–**d**) Refractive index sensing: Analyte infiltration increases the effective optical thickness, causing a redshift in the interference spectrum (Reflectance mode). (**e**,**f**) Photoluminescence sensing: Surface interactions modulate non-radiative recombination, leading to intensity quenching [84].

Optical sensing represents a primary application domain where the structural versatility of porous silicon (PSi) is directly translated into functional device performance. The fundamental transduction mechanism, systematically visualized in Figure 10 [84], relies on the specific functionalization of the pore walls and the subsequent infiltration of analytes into the silicon matrix (Figure 10a). This accumulation of molecular matter increases the effective refractive index (n_eff_) of the porous medium, which serves as the physical basis for signal modulation. For interferometric devices such as Fabry–Pérot thin films, the optical response is governed by the effective optical thickness (EOT ≈ 2n_eff_L). In a typical reflectance spectroscopy setup (Figure 10b), broad-spectrum light illuminates the sample, and the reflected interference pattern is analyzed. Analyte binding increases the optical path length, manifesting as a distinct redshift of the Fabry–Pérot interference fringes (Figure 10c). To strictly quantify this change, the spectral data is processed via Fast Fourier Transform (FFT), converting the periodic interference signal into a single peak representing the optical thickness; sensing is thus recorded as a positive shift in the EOT peak position (Figure 10d). Distinct from refractive index-based interrogation, transduction can also leverage the intrinsic electronic properties of the silicon skeleton via photoluminescence (PL) spectroscopy (Figure 10e). In this mode, surface interactions typically modify the non-radiative recombination centers on the silicon nanocrystallites, resulting in a measurable quenching of the PL intensity (Figure 10f). These complementary mechanisms enable label-free, real-time monitoring, provided that the optical response is effectively decoupled from mass transport limitations [84].

Traditionally, PSi sensors have relied on “flow-over” configurations, which often suffer from diffusion-limited response times. To address this, recent advances have focused on “flow-through” architectures that integrate seamlessly with planar microfluidics. He et al. developed a lateral porous silicon membrane transducer that facilitates in-plane fluid flow, effectively transforming the device into an on-chip Fabry–Pérot interferometer. This structural engineering allows for flow-through operation without compromising interferometric quality. In refractive index sensing applications, this lateral architecture demonstrated a sensitivity exceeding 150 nm·RIU^−1^ and suppressed the limit of detection (LOD) to below 10^−3^ RIU, proving that pore orientation engineering can significantly enhance integrability and quantitative performance while maintaining the material’s intrinsic sensing capabilities [85].

When transitioning from bulk refractive index sensing to specific biomolecular recognition, the limiting factor shifts from optical sensitivity to reaction kinetics and mass transport. Awawdeh et al. highlighted this interplay in a PSi Fabry–Pérot aptasensor for lactoferrin detection. By systematically optimizing the pore structure, layer thickness, and capture probe density, the authors first established a baseline LOD of 50 nM. However, by integrating the sensor into a 3D-printed microfluidic system featuring active micromixing, this LOD was improved by over an order of magnitude. This result underscores a critical design philosophy: the optical mode transduces the pore occupancy, but the ability to reach low detection limits is governed by engineering the fluid dynamics to replenish the depletion layer at the pore interface [79]. Notably, recent automated systems driven by rotating magnetic fields have achieved ‘sample-in–answer-out’ capabilities [86], offering a blueprint for portable diagnostics.

While spectral shifts provide rich data, intensity-based readouts offer a pathway toward simpler, cost-effective instrumentation suitable for field-deployable devices. Zhang et al. exploited the steep spectral slope at the bandgap edge of a PSi Bragg mirror to facilitate high-sensitivity detection using a single-wavelength source (633 nm). By adjusting the incidence angle to a “dark” operating point, the system converts minute refractive index changes into significant variations in reflected light intensity, quantifiable via grayscale analysis. This approach achieved an LOD as low as 20.74 pM for DNA detection, while maintaining a linear response within the 0.1–5 nM concentration range, demonstrating that the high-Q features of photonic crystals can be leveraged to enable low-cost imaging-based readouts without sacrificing analytical rigor [87]. However, for field-deployable imaging devices, environmental interference remains a challenge. Recent advances in adversarial spatio-temporal learning for video deblurring [88] and adaptive degradation-aware models for restoring weather-degraded images [89] suggest that integrating advanced computational imaging algorithms can significantly enhance signal fidelity. Furthermore, combining these optical sensors with UAV-based platforms utilized for quantitative structural measurement [90] could expand the operational range of PSi sensors to remote infrastructure monitoring.”

To further push the limits of sensitivity, optical transduction is increasingly coupled with biochemical amplification and hybrid physics strategies. This strategy is not only applicable to toxins like Aflatoxin B1 but also aligns with metabolomic profiling methods used in food quality assessment, such as analyzing the flavor and antioxidant profiles of pear soup [91]. Nanda Kumar et al. utilized this strategy for the detection of Aflatoxin B1 in agricultural crops, employing a competitive immunoassay format to achieve a broad dynamic range of 0.01–10 ppb and an LOD as low as 0.03 ppb [92].

Furthermore, the field is expanding toward multiphysics platforms that combine photonic confinement with plasmonic enhancement. Hoang et al. developed a hybrid Surface-Enhanced Raman Scattering (SERS) substrate by decorating PSi microcavities with silver nanoparticles (AgNPs). This architecture benefits from the dual enhancement of the AgNP localized surface plasmon resonance (LSPR) and the photon trapping effect of the microcavity. The platform demonstrated ultrasensitive detection of Rhodamine 101 at 1.0 × 10^−13^ M and methyl parathion at 1.0 × 10^−10^ M, while maintaining excellent signal uniformity (RSD ≈ 6.14%) and long-term stability (>50% signal retention after 28 days) [93]. These advances illustrate that PSi is evolving from a passive optical transducer into a versatile platform where structural, chemical, and optical properties are engineered in concert to solve complex sensing challenges.

### 4.2. Chemical Sensors: Detection of VOCs, Humidity, and Toxic Gases

**Figure 11:**
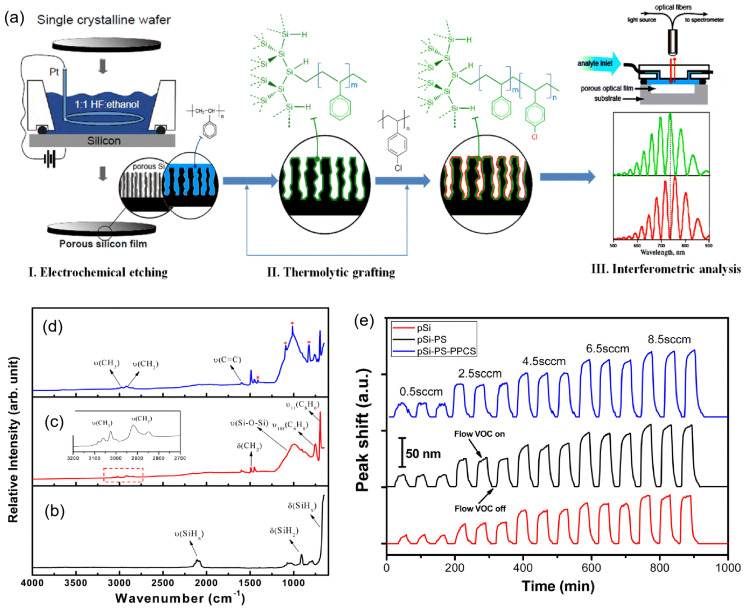
Enhanced VOC selectivity via double-grafted surface chemistry. (**a**) Fabrication workflow illustrating electrochemical etching, sequential thermolytic grafting of polystyrene (PS) and poly(4-chlorostyrene) (PPCS), and the interferometric analysis setup (blue arrows indicate the process sequence); (**b**) FTIR spectrum of the as-prepared hydride-terminated pSi; (**c**) FTIR spectrum of the single-grafted pSi-PS; (**d**) FTIR spectrum of the double-grafted pSi-PS-PPCS; (**e**) Real-time interferometric response comparing the peak shift behavior of pristine pSi (red), pSi-PS (black), and pSi-PS-PPCS (blue) under varying VOC concentrations [103].

The chemical sensing capabilities of porous silicon (PSi) derive from its unique combination of tunable pore architecture, high specific surface area, and versatile surface chemistry. Unlike metal oxide semiconductor (MOS) sensors, the response in PSi is typically governed by a complex coupling of intra-pore mass transport, surface adsorption/reaction, and carrier or dielectric polarization. To address this complexity, recent strategies have focused on “locking” the response to predictable dominant mechanisms through structural engineering—such as the use of hierarchical or rugate pores—and interface modification via oxidation, organic grafting, or inorganic capping. This need for sophisticated signal discrimination is particularly acute in volatile organic compound (VOC) detection, where PSi exhibits a generalized response to most organic vapors. Consequently, the field has moved beyond single-parameter measurements toward multidimensional fingerprinting. To decode these complex signals against environmental noise, researchers are adapting advanced algorithmic frameworks from data-intensive engineering domains. For instance, dynamic-constrained digital twins [94] and diffusion-model-based diagnostics [95] offer robust mathematical paradigms for modeling non-linear sensor drift, while temporal-attention mechanisms [96] and multi-rate data fusion strategies [97] allow for the precise extraction of features from noisy time-series data. Building on these foundations, the integration of dimension-reduction optimization [98], intelligent fault diagnosis schemes [99], and predictive classification algorithms [100] is transforming raw impedance or optical data into reliable, real-time chemical decisions. Zhou and Sohn pioneered this by coupling reflectance spectroscopy with photoluminescence (PL) on rugate filters, demonstrating that while single parameters may overlap, the 2D “shift-intensity” trajectories are distinct. For instance, while hexane and chloroform induced comparable reflectance redshifts (~58 nm and 53 nm), their PL quenching behaviors were vastly different (11.4% vs. 52.7%), allowing for clear discrimination [101]. Expanding this concept spatially, Chun and Miskelly utilized hyperspectral imaging on rugate chips featuring side-by-side oxidized and methylated zones, effectively encoding chemical affinity into spatial patterns for concentration-independent solvent identification [102]. Further structuring the response via rational interface design, Vo et al. demonstrated a surface grafting strategy that establishes a reproducible thermodynamic sensitivity hierarchy, as illustrated in Figure 11. By employing a facile non-atmospheric thermolysis process, the authors first grafted styrenic carbon fragments onto the pore walls, followed by a second thermolysis step to graft poly(4-chlorostyrene) (PPCS), resulting in a double-grafted pSi-PS-PPCS composite (Figure 11a). As evidenced by the dynamic peak shift response (Figure 11e), this double-grafted sensor exhibited significantly higher sensitivity compared to both pristine pSi and single-grafted pSi-PS. This enhancement was attributed to strong intermolecular interactions—specifically hydrophobic, hydrogen bonding, and π-π stacking—between the analytes and the chlorine-substituted phenyl moieties of the grafted PPCS. Consequently, the sensor displayed a distinct sensitivity order (n-hexane < ethanol < isopropanol < M.E.K. < isobutanol < ethyl acetate), effectively translating chemical affinity into a quantifiable optical metric [103]. Finally, addressing long-term reliability, Zhang et al. developed ionic liquid-infiltrated PSi arrays that generate robust cross-reactive patterns with shelf stability exceeding 1.5 years [104]. Collectively, these works illustrate that VOC sensing is evolving into a pattern recognition science driven by photonic amplification and multi-parameter data fusion.

While VOC sensing often exploits physical condensation patterns, humidity sensing is dominated by the formation of chemisorbed water layers and subsequent capillary condensation, which modulate the dielectric constant and facilitate charge transport via the Grotthuss mechanism. Consequently, to ensure reliable readout in this regime, the stabilization of hydrophilic interfaces is paramount to minimize hysteresis and baseline drift. Jung and Yoo demonstrated this mechanism using freestanding porous SiO_2_/Si films, where impedance spectroscopy confirmed that the capacitive response was primarily driven by proton hopping transfer between adjacent hydroxyl groups within the chemisorbed water layer. Operating within a range of 13.8–79.0% RH, their device exhibited response/recovery times of approximately 18 s/30 s and a hysteresis error of ~5.0%, underscoring that a continuous hydrogen-bonding network on a stable oxide surface is essential for reproducible electrical readout [105]. Validating the engineering aspect of this “hydrophilic pinning” strategy, Park et al. quantified the impact of inorganic encapsulation by comparing hydrophobic MACE-etched PSi (contact angle 137.6°) with hydrophilic ALD-coated porous Si_3_N_4_ structures (contact angle 15.3°). The latter achieved linear capacitive response (R^2^ = 0.998) across 18.48–81.67% RH with rapid dynamics (13.3 s/12.4 s), effectively locking the interface into a state where capillary condensation and surface conduction channels are the sole variables [106]. This stability enabled deployment even in alkaline cement matrices. As highlighted in recent studies on functional material modified concrete [107], monitoring the internal moisture and crack resistance of construction materials is critical, and inorganic-capped PSi sensors offer a viable solution. Further emphasizing the tunability of surface states, Wan Ahmad Aziz et al. systematically investigated the effect of thermal annealing on sensitivity. In their Au/PSiO_2_/Au resistive sensors, annealing at 450 °C optimized the density of hydrophilic Si–O–Si/Si–OH sites, resulting in a peak sensitivity of ~18.5 μA/%RH (at 10 V bias). This result reinforces the conclusion that thermal oxidation serves as a controllable knob to maximize water molecule interaction at active sites while stabilizing the output [108]. Finally, extending this logic to optical readout, Abdul Hussien et al. demonstrated that surface modification with reduced graphene oxide (rGO) enhances the photoluminescence (PL) response to humidity. While the transduction mode differs, the underlying physics remains consistent: the adsorption and subsequent condensation of water molecules modulate the local dielectric environment and recombination dynamics, further illustrating that the “adsorption-condensation” mechanism is the universal driver for humidity sensing across different readout modalities [109].

In contrast to the dielectric mechanisms dominating humidity sensing, the detection of toxic gases such as nitrogen dioxide (NO_2_, an electron acceptor) and ammonia (NH_3_, an electron donor) typically relies on a coupled process: adsorption-induced charge transfer combined with the modulation of carrier transport within the porous network. Consequently, performance optimization requires the simultaneous engineering of intra-pore mass transport channels and surface defect/oxidation states to facilitate efficient carrier exchange. For NO_2_ detection, this structure–property relationship has been validated through various surface modification strategies. Mhamdi et al. established the baseline feasibility using stain-etched PSi, achieving room-temperature detection down to 4 ppm with a response time of approximately 45 s, demonstrating that even low-cost fabrication routes can support viable charge-transfer readout [110]. To further enhance efficiency, recent work has focused on the synergistic effect of oxygen-rich surfaces and open pore architectures. Hayif et al. employed a non-thermal atmospheric pressure argon plasma jet (18 kV) to modify the PSi surface, which drastically altered the morphology and chemistry: pore diameter expanded from 1.1 to 2.4 μm, porosity increased from 11% to 75%, and oxygen content rose from 0.3% to 21.1%. This modification resulted in a maximum sensitivity to 60 ppm NO_2_ at 75 °C, effectively coupling improved mass transport with an increased density of active sites [111]. Moving toward rigorous quantification and stability, Qiang et al. constructed a Pd-modified V_2_O_5_/PSi composite. This system demonstrated a systematic response to NO_2_ in the 0.25–2 ppm range at 25 °C, with response values scaling from 1.5 (at 0.25 ppm) to 5.6 (at 2 ppm) and rapid kinetics (4.5 s response). Crucially, the sensor maintained long-term stability with fluctuations controlled within ±3.75% over 30 days, proving that the introduction of catalytic phases can transform PSi from a merely responsive material into a calibratable, robust sensor [112].

For ammonia (NH_3_) sensing, the same logic applies. Performance depends on optimizing surface states for electron/hole exchange and designing device structures that convert this exchange into stable electrical signals. Khaniyev et al. demonstrated that electrochemically etched PSi alone possesses sufficient intrinsic sensitivity to achieve sub-ppm (<0.1 ppm) detection with high linearity at room temperature [113]. However, the magnitude of the resistive output is strongly influenced by electrode geometry. Ali et al. compared different configurations on macroporous silicon, reporting that a sandwich electrode structure enhanced the gas response to 3.5, compared to 1.85 for a planar configuration, while also improving response/recovery dynamics [114]. For more aggressive engineering of reaction kinetics, Almansba et al. utilized a CuO/Co_3_O_4_/PSi thin-film heterojunction. This composite achieved a 200% response at 170 ppm with ultra-fast dynamics (3 s/1 s response/recovery) without the need for heating, highlighting that heterojunction design can effectively manipulate depletion layer behavior for second-level response times [115]. Mechanistically, this mirrors the atomic-level charge flow steering and S-scheme heterojunction dynamics observed in advanced photocatalysts [116,117]. Similarly, in the broader context of porous nanomaterials, porous ceria hollow microspheres have demonstrated remarkable sensitivity in electrochemical detection [118]. For practical deployment, sensors must also adapt to dynamic urban environments, such as monitoring air quality in metro systems [119] or integrating with the high-speed data streams [120] to ensure passenger safety. To fully realize this potential in dynamic urban networks, sensors must evolve into intelligent nodes capable of secure data transmission. Integrating PSi sensors with secure distributed computing architectures—such as federated deep learning frameworks [121] and augmented AIoT systems [122]—enables privacy-preserving collaborative learning across sensor arrays. This allows for real-time pollution mapping without compromising data security, bridging the gap between individual device sensitivity and city-scale environmental monitoring. Finally, addressing the critical issue of cross-sensitivity between these polar gases and humidity, Baratto et al. established a methodological framework using impedance spectroscopy on free-standing PSi membranes. By analyzing relaxation constants, they demonstrated that selecting specific frequency windows allows for the effective decoupling of the gas-induced charge transfer signals from the dielectric contributions of water vapor, providing a robust path for practical deployment in ambient environments [123].

To contextualize the commercial viability of porous silicon chemical sensors, it is necessary to benchmark their performance against competing material platforms such as metal oxide semiconductors (MOX), carbon nanotubes (CNTs), and conductive polymers. While widely used MOX sensors generally offer superior sensitivity, they necessitate high operating temperatures (>200 °C), resulting in significant power consumption. In contrast, porous silicon enables room-temperature operation with unique advantages in monolithic CMOS integration, albeit with trade-offs in response dynamics governed by pore diffusion. A detailed quantitative comparison of these technologies—focusing on sensitivity, selectivity, response/recovery time, stability, and cost—is presented in Table 1.

### 4.3. Biosensing: DNA, Proteins, and Pathogens

**Figure 12:**
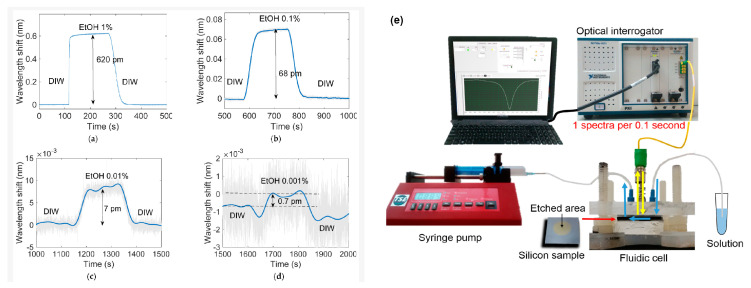
Limit-of-detection analysis in a microfluidic porous silicon microcavity. (**a**) Real-time sensorgram resolving a 620 pm spectral shift for 1% ethanol. (**b**) Sensorgram for 0.1% ethanol showing a 68 pm shift. (**c**) Sensorgram for 0.01% ethanol showing a 7 pm shift. (**d**) Sensorgram for 0.001% ethanol resolving a 0.7 pm shift, defining the detection limit; the grey dashed lines indicate the peak-to-peak signal amplitude relative to the baseline noise floor. (**e**) Integrated microfluidic setup for rigorous kinetic measurements; the red arrow points to the etched sample area, blue arrows show the solution flow direction, and yellow arrows denote the optical interrogation paths [126].

In biosensing scenarios, porous silicon (PSi) functions simultaneously as a “nanoporous reaction vessel” and an “optical resonator.” The surface-immobilized biorecognition elements—such as DNA probes, antibodies, peptides, or glycoproteins—govern the selectivity, while photonic structures like Fabry–Perot interferometers, Distributed Bragg Reflectors (DBRs), and microcavities amplify local refractive index variations into resolvable spectral shifts or colorimetric changes. Unlike the chemical sensing discussed previously, bioanalysis typically involves high-molecular-weight targets (nucleic acids, proteins, bacteria). Consequently, the sensor’s ultimate sensitivity and reproducibility are critically dependent on the targets’ infiltration into the pore channels, their binding kinetics, and the stability of the functional interface. For nucleic acid detection, recent advancements have transitioned from simple single-layer interferometric structures to high-quality factor (Q) PSi optical microcavities coupled with silicon-based integrated platforms. Zhang et al. fabricated a PSi microcavity biosensor on a silicon-on-insulator (SOI) wafer designed for an operating wavelength of 1555 nm. By precisely engineering the refractive index and thickness of the bilateral DBRs and the central defect layer, the authors achieved a narrow resonance peak with a full width at half maximum (FWHM) of approximately 26 nm. In hybridization experiments involving 19-base pair DNA targets, the resonance redshift exhibited excellent linearity against complementary strand concentrations in the 0.625–12.5 μM range, ultimately achieving a limit of detection (LOD) of 43.9 nM. This demonstrates that PSi microcavities constructed on SOI platforms can realize sub-100 nM label-free DNA detection while maintaining compatibility with CMOS processes [124]. Addressing the mass transport limitations inherent in deep pores, similar concepts have been extended to “assembled” microcavity structures. By mechanically assembling a PSi DBR with a separately prepared microcavity unit, researchers achieved label-free detection of short-chain DNA with an LOD of 22.5 nM, noting that this modular approach significantly mitigates issues related to biomolecular infiltration and incomplete washing [125]. Building on these architectures to establish a rigorous performance benchmark, Caroselli et al. coupled high-sensitivity PSi microcavities with a custom microfluidic system to systematically evaluate the refractive index response under continuous flow. As illustrated in Figure 12e, the setup utilized a high-speed optical interrogator (10 Hz sampling rate) to monitor resonance shifts while a syringe pump delivered analyte solutions. By cycling ethanol concentrations ranging from 1% down to 0.001% against a deionized water baseline, the device resolved distinct spectral steps, measuring wavelength shifts from 620 pm down to a minute 0.7 pm (Figure 12a–d). This ability to distinguish sub-picometer drifts against the signal noise confirms a minimum resolvable refractive index change on the order of 10^−7^ RIU. These empirical results validate a high sensitivity of ~1000 nm·RIU^−1^, defining the “refractive index noise floor” essential for ultra-low concentration biomolecule detection [126].

In the domain of protein analysis, the focus shifts toward amplifying these refractive index signals via functional assays. Krismastuti and Voelcker et al. exploited porous silicon resonant microcavities (pSiRM) embedded with fluorescent peptide substrates for the ultrasensitive detection of matrix metalloproteinase (MMP) activity. The immobilization of MMP-cleavable fluorescent peptides on the inner cavity walls allowed the enzymatic reaction to simultaneously alter the pore medium’s refractive index and modulate the intracavity field distribution. This dual mechanism significantly amplified both the fluorescence signal and the resonant spectral shift, yielding a remarkable LOD of 7.5 × 10^−19^ M within a short 15 min detection window, highlighting the potential for rapid wound healing monitoring [127]. Furthermore, Wei et al. integrated PSi Bragg reflectors with quantum dot (QD) labeling to construct a dual-signal detection platform for -lactoglobulin. By digitally capturing both the intensity variations at the reflectance edge and the QD fluorescence, the platform achieved an LOD of 0.12 ng·mL^−1^ [128]. While not strictly “label-free,” the refractive index amplification and field enhancement provided by the photonic structure remain the core physical basis for its ultra-high sensitivity.

Beyond molecular targets, the high specific surface area and programmable surface chemistry of PSi have been adapted for the analysis of bacteria and complex disease markers. The Lectin-coupled PSi system provides a definitive example of label-free bacterial differentiation; a 2020 study utilized reflective interferometric Fourier transform spectroscopy (RIFTS) on lightly oxidized/silanized PSi films grafted with lectins to produce distinguishable spectral redshifts within the 10^3^–10^5^ CFU·mL^−1^ concentration range [81]. Principal Component Analysis (PCA) of these shifts enabled the discrimination of Escherichia coli and Staphylococcus aureus, proving that rational matching of surface glycan recognition with optical readout can achieve bacterial Gram-typing without fluorescence. Parallel progress has been made using high-Q modes for specific disease marker diagnosis. For example, Zhang et al. developed a disposable peptidoglycan-specific biosensor capable of noninvasive real-time detection of broad-spectrum Gram-positive bacteria [129]. Krismastuti et al. developed pSiRM arrays functionalized with fluorescent peptide substrates targeting S. aureus Sortase A (SrtA), enabling the parallel identification of multiple wound biomarkers at low nanomolar concentrations on a single chip [130]. Addressing major public health issues such as parasitic diseases, Li and coworkers utilized near-infrared transmission angle spectroscopy for the detection of the 43 kDa antigen associated with hydatid disease [131]. By measuring the shift in the incident angle required for maximum transmission through the PSi microcavity, the antigen-antibody reaction-induced RI change was converted into an angular shift signal, avoiding the need for high-resolution spectrometers while maintaining high sensitivity suitable for rapid screening. In summary, the PSi optical microcavity has evolved from a simple refractive index sensing unit into a unified diagnostic tool for multi-scale targets, where the integration of SOI-compatible structures and high-Q modes significantly lowers detection limits and shortens analysis times.

To rigorously assess the translational potential of porous silicon biosensors, it is necessary to benchmark their performance against established clinical tools and emerging nanomaterials. While Surface Plasmon Resonance (SPR) serves as the gold standard for label-free kinetics, its high instrumentation cost and planar binding geometry limit point-of-care deployment. Conversely, while ELISA offers high specificity, it remains restricted by labor-intensive, labeled workflows. Porous silicon bridges this gap by offering 3D volumetric binding and label-free readout compatible with standard CMOS manufacturing. A detailed comparative analysis of these technologies, focusing on limits of detection, label requirements, and integration capabilities, is presented in Table 2.

## 5. Advances in Energy Storage

### 5.1. Lithium-Ion Batteries (LIBs) Anodes

The fundamental rationale for employing porous silicon (PSi) in lithium-ion battery anodes lies in utilizing its intrinsic porosity as an “internal reserve” to accommodate the immense volumetric expansion (>300%) of silicon during lithiation. By simultaneously shortening the diffusion paths for Li-ions and electrolytes via interconnected channels, PSi establishes a tunable window between cycling stability and rate capability. However, as the objective shifts from merely achieving cycle life to engineering high-energy-density cells, the structural advantages of PSi are challenged by pragmatic constraints in grid-level applications, such as stabilizing offshore wind energy [132] and responding to AI-based load forecasting [133]: high specific surface area, which exacerbates initial coulombic efficiency (ICE) losses and continuous side reactions; and inherent porosity, which compromises tap density and calendering resistance, thereby diminishing volumetric energy density and narrowing the electrode processing window. Consequently, recent advancements have pivoted from simply maximizing porosity toward a synergistic design of “pore structure–interface–compaction.” This design draws on microstructural tuning strategies from alloys [134], soft/hard interfaces in MXenes [135,136], and advanced solvent principles [137].

**Figure 13:**
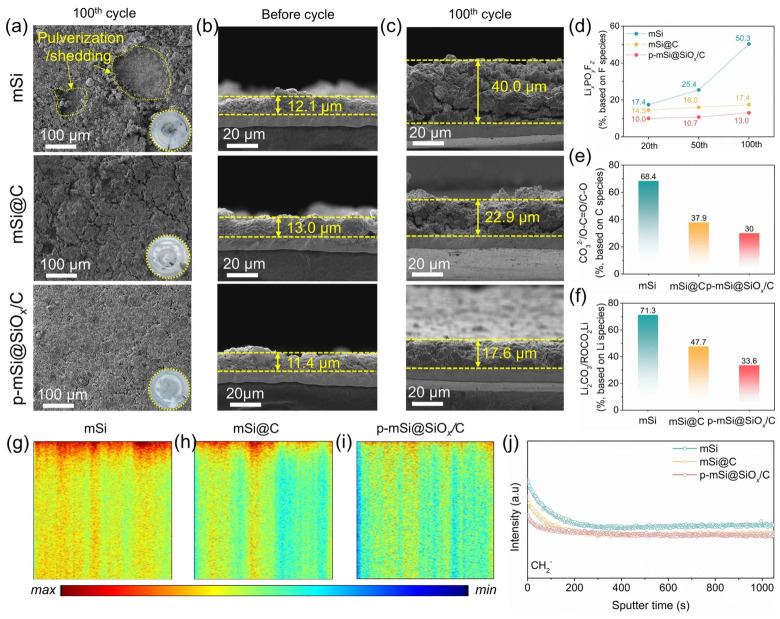
Structural integrity of engineered porous silicon anodes. (**a**) Surface SEM image of the unmodified mSi electrode after 100 cycles, where yellow circles highlight regions of severe pulverization and particle shedding; (**b**) cross-sectional SEM of the cycled mSi electrode demonstrating large thickness expansion; (**c**) SEM image of the engineered p-mSi@SiOx/C electrode after cycling, showing suppressed expansion and maintained structural integrity; (**d**–**j**) chemical analysis via XPS and TOF-SIMS confirming that the stable composite interface minimizes electrolyte decomposition and SEI thickening [138].

To address the bottleneck of low volumetric density in nanostructured silicon, micron-scale engineering for high tap density has emerged as a representative strategy. Liu et al. utilized a hydrolysis-driven approach to synthesize micron-sized porous Si, forming a continuous SiOx/C composite layer on both internal and external surfaces. The effectiveness of this architecture is first evidenced by its high tap density of approximately 0.65 g·cm^−3^, while quantitative mechanical analysis further confirmed that the average stress in the fully lithiated state was limited to a low level of ~9.94 GPa. This mechanical robustness translates directly into superior structural integrity during cycling, as visually confirmed in Figure 13. While unmodified micron-sized Si (mSi) electrodes suffered from severe “pulverization/shedding” and uncontrolled volume expansion—swelling from an initial ~12.1 μm to 40.0 μm after 100 cycles (Figure 13a–c)—the engineered p-mSi@SiOx/C electrode maintained a dense, cohesive morphology with electrode thickness increasing only marginally to 17.6 μm. Furthermore, surface analysis via TOF-SIMS and XPS (Figure 13d–j) revealed that the composite coating effectively suppressed electrolyte permeation and stabilized the solid electrolyte interphase (SEI), resulting in a thinner, inorganic-rich passivation layer compared to the thick, carbonate-rich accumulation found on mSi. Electrochemically, the material retained 901.1 mAh·g^−1^ after 500 cycles at 1 A·g^−1^ and delivered rate capacities of 1123.0 and 850.4 mAh·g^−1^ at high current densities of 5 and 8 A·g^−1^, respectively. Crucially, this work successfully translated structural buffering into cell-level performance: achieving an areal capacity of 2.09 mAh·cm^−2^ over 100 cycles in a pouch cell paired with an NCM811 cathode, illustrating that micron-porous Si possesses the potential to satisfy rigorous engineering evaluation criteria through improved compactibility and mass loading capabilities [138].

Simultaneously, interfacial catalysis is being introduced to regulate the SEI. Drawing inspiration from advanced Li-S battery catalysts [139,140] and MOF-derived kinetics studies [141], this strategy aims to transform the PSi interface into an active field for solid electrolyte interphase (SEI) regulation, thereby mitigating degradation caused by repeated ruptures, while structural integrity is reinforced by insights from carbon evolution [142,143] and steel toughening mechanisms [144]. For instance, Liu et al. constructed a defect-rich amorphous oxide layer (alumina–titanium oxide) in situ on the PSi surface. This functional layer facilitated the selective adsorption and catalytic decomposition of fluoroethylene carbonate (FEC), inducing the formation of a robust, LiF-enriched SEI. The resulting anode exhibited remarkable stability, retaining 79.8% capacity after 1000 cycles at 5 A·g^−1^, and even more impressively, it delivered 692 mAh·g^−1^ at an ultra-high rate of 25 A·g^−1^ and maintained 80.0% retention after 500 cycles at 50 °C. This approach elevates the role of porosity from simple mechanical buffering to a platform for programmable interfacial chemistry, where SEI stability directly dictates the reversibility of high-density reaction fronts [145].

In parallel, to balance cost with performance, the construction of scalable feedstocks and conductive networks has become another critical trajectory. Sun et al. derived micron-sized porous Si (μP-Si) from waste AlSi alloys, constructing a “tough composite interface network” via CNT coating and PVP-derived carbon. This μP-Si@CNT@C composite achieved a reversible capacity of approximately 3500 mAh·g^−1^ at 0.2 A·g^−1^, validating the efficacy of scalable synthesis and the closed-loop design of “structure–conductivity–interface toughness [146].” Similarly, Li et al. utilized photovoltaic kerf loss silicon to fabricate pSi/Ag@C via Ag-assisted chemical etching and tannic acid carbonization. With an embedded silver content of ~3.3 wt%, this composite retained 665.3 mAh·g^−1^ after 100 cycles at 0.5 A·g^−1^ and demonstrated robust rate performance, delivering 2369.5, 2015.1, 1635.7, and 1033.8 mAh·g^−1^ at 0.1, 0.25, 0.5, and 1.0 A·g^−1^, respectively [147]. These strategies indicate that by utilizing low-cost precursors and embedding electronic transport and structural integrity into the porous matrix, one can effectively respond to the engineering constraints of practical applications.

It is worth noting that porosity does not automatically translate to longevity; thus, surface area management and electrode integration are key dimensions determining final performance. As excessive surface area consumes Li-ions and electrolytes, reducing the effective external surface area while maintaining internal buffering is paramount. A “polymer sealing” strategy provides a clear quantitative example: by creating a cross-linked conductive polyaniline (c-PANi) coating, a closed pore volume of 1.49 cm^3^·g^−1^ was successfully encapsulated, significantly reducing the specific surface area to 5.7 m^2^·g^−1^ [148]. This “internal/external division of labor”—where internal pores buffer volume and external interfaces inhibit SEI growth—significantly improved ICE and long-term coulombic efficiency. At the electrode level, Güneren et al. demonstrated the importance of binder chemistry in a Si/graphite (20:80) system: a functionalized alginate binder containing sulfonate groups (s-Alg) exhibited lower electrolyte uptake (11.5%) and higher tensile strength (97 MPa), which directly contributed to a high initial electrode capacity of 1250 mAh·g^−1^ and a ~65% improvement in capacity retention compared to CMC/SBR at 0.2 C [149]. Ultimately, techno-economic analyses of magnesiothermic reduction (MgTR) routes suggest that, under specific assumptions, PSi anodes can approach the cost-competitiveness of graphite, further confirming that the path to commercialization relies on balancing performance metrics with raw material sustainability and electrode processability.

### 5.2. Supercapacitors

The utilization of porous silicon (PSi) in supercapacitors extends beyond leveraging its “high specific surface area”; fundamentally, it provides a 3D porous framework capable of in situ fabrication on silicon substrates. While this architecture minimizes ion diffusion lengths and maximizes the effective interfacial area, it simultaneously exposes critical material bottlenecks: insufficient electronic conductivity of the silicon skeleton, surface chemical instability (susceptibility to oxidation/dissolution), and challenges in deep-pore wetting and equivalent series resistance (ESR) control. Consequently, recent research has converged toward a clear engineering strategy: first, establishing a stable electric double-layer capacitor (EDLC) baseline using conductive/barrier layers (e.g., TiN) to ensure electrical contact and chemical pinning, followed by the integration of transition metal oxides or conductive polymers to introduce fast, reversible Faradaic reactions for pseudocapacitive gain.

In EDLC-dominated systems, the primary strategy involves treating PSi as a scalable 3D electrode scaffold and converting it into a functional capacitive interface via process-compatible thin films. Isakov et al. fabricated on-chip micro-supercapacitors based on “grass-like alumina nano-electrodes (GLA) on porous silicon,” employing ALD-deposited TiN/GLA/TiN layers to achieve conformal conductivity and interfacial stability. This device delivered an areal specific capacitance of 5–11 mF·cm^−2^ and a volumetric energy density of approximately 12 μWh·cm^−3^, retaining ~95% of its capacity after 10,000 cycles. Notably, the self-discharge behavior showed a voltage drop from 0.64 V to ~0.48 V after 20 h (≈25% loss), highlighting the effectiveness of the “conductive network + barrier layer” in constraining leakage pathways [150]. To address the poor wettability and high internal resistance inherent in high-aspect-ratio PSi matrices, Grigoras et al. extended this conformal coating strategy by depositing a ~10 nm TiN layer via ALD throughout the deep pore channels. This passivation ensured continuous electrical contact and electrolyte accessibility, translating into a high volumetric capacitance of 15 F·cm^−3^ and an energy density of 1.3 mWh·cm^−3^ [151]. Furthermore, the robust interface enabled a power density of 214 W·cm^−3^ and stability exceeding 13,000 cycles. Beyond capacity, when targeting ultra-high frequency response, the synergy between electronic and ionic transport becomes decisive [151]. Yao et al. demonstrated that by combining high-conductivity TiN (3.67 × 10^4^ S·m^−1^) with a hierarchical pore structure, devices could operate at scan rates up to 100 V·s^−1^ with a characteristic time constant of only 4 ms and zero capacitance decay over 200,000 cycles [152]. Collectively, these findings underscore that realizing full pore-wall electrical contact and mass transport accessibility is often more critical for EDLC performance than simply increasing geometric surface area.

When the research objective shifts from material feasibility to CMOS-compatible integration (i.e., on-chip applications), the role of compatible materials like TiN or NiO becomes even more pronounced. Thamri and Raouadi fabricated NiO/PS/Si micro-supercapacitors by passivating a stain-etched PSi layer (pore size ~47 nm, depth ~450 nm) with sol–gel NiO. This functionalization yielded drastic improvements: the effective series resistance dropped from 1.8 Ω·cm^2^ (NiO/Si) to 0.05 Ω·cm^2^ (NiO/PS/Si), while areal capacitance increased from 12.34 μF·cm^−2^ to 9.64 mF·cm^−2^. The device maintained ~97% of its initial capacitance after 5000 cycles (0–0.8 V) with an energy efficiency of ~91% [153]. Progressing toward robust solid-state integration, Jhajhria et al. demonstrated that “conformal conductive/barrier layers” are essential base engineering rather than optional modifiers. By using sputtered TiN to passivate p-type macroporous Si (pore length ~9 μm, width ~3–4 μm) and coupling it with a Na_2_SO_4_–PVA hydrogel, they achieved an all-solid-state device operating within a wide 2 V window, yielding an areal capacitance of 8.6 mF·cm^−2^ and an energy density of 1.96 μWh·cm^−2^ at 0.1 mA·cm^−2^ [154]. This demonstrates that in on-chip architectures, a tripartite division of labor—PSi for 3D ion channels, surface films for electronic pathways/stability, and active phases for pseudocapacitance—is more effective than simply pursuing higher porosity.

**Figure 14:**
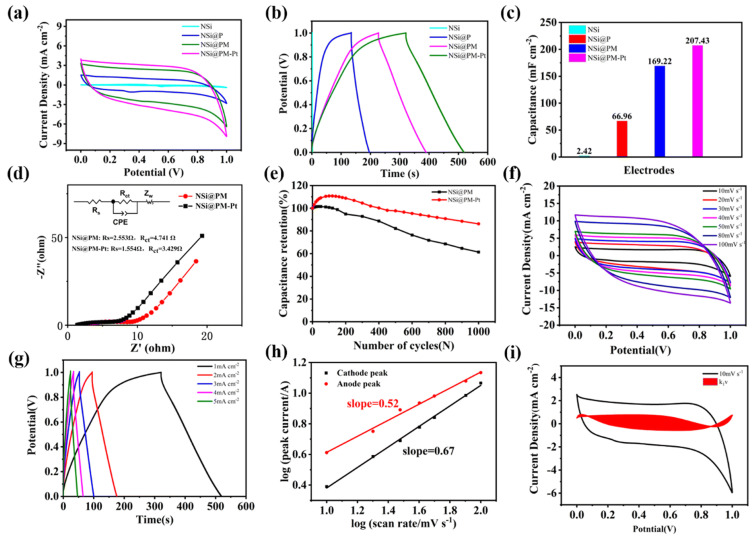
Electrochemical performance of the hierarchical SiNWs/Ni/PEDOT–MnOx electrode. (**a**–**e**) Cyclic voltammetry and charge–discharge profiles show enhanced capacitance (207.43 mF cm^−2^) and stability compared to non-conductive controls. (**f**–**i**) Kinetic analysis validates a mixed charge storage mechanism dominated by pseudocapacitive surface reactions, supported by low charge transfer resistance [156].

Beyond physical adsorption, to convert the high interfacial area of PSi into reversible charge storage and realize pseudocapacitive gain, the integration of transition metal oxides and conductive polymers requires a robust conductive skeleton to ensure interfacial stability. In inorganic systems, Ge et al. first established that chemical stability and conductive pathways are prerequisites for converting “theoretical surface area” into measurable capacitance; by compositing ZnO nanoparticles with PSi, they reported a specific capacitance of 3.9 mF·g^−1^—a 40-fold increase over pristine PSi—attributed primarily to ZnO-mediated pore wall passivation and improved charge transport [155]. Building on this necessity for interfacial engineering, Liu et al. [156] implemented a “protection-plus-collection” strategy by electrodepositing Ni particles on Si nanowires (SiNWs) to support a PEDOT–MnOx composite. The comprehensive electrochemical evaluation in Figure 14 validates this hierarchical design. As shown in Figure 14a,b, the stepwise functionalization (from NSi to NSi@PM-Pt) results in a progressive expansion of the CV hysteresis loop and a significant prolongation of the GCD discharge time, culminating in a maximized areal capacitance of 207.43 mF·cm^−2^ (Figure 14c). This performance enhancement is mechanistically supported by Electrochemical Impedance Spectroscopy (Figure 14d), which reveals that the conductive Ni interlayer reduces the charge transfer resistance (Rct) from 4.741 Ω to 3.429 Ω, effectively lowering the interfacial barrier. Furthermore, kinetic analysis (Figure 14h,i) indicates that the optimized electrode operates with mixed diffusion-controlled and capacitive mechanisms (b-values of 0.52/0.67), maintaining robust rate capability across varying scan rates (Figure 14f) and current densities (Figure 14g). This stable architecture translates into superior durability, showing significantly improved capacity retention compared to the non-conductive control (Figure 14e), with long-term testing confirming 94.6% retention after 5000 cycles [156]. Further optimizing this tripartite division of labor, the authors demonstrated that embedding MnOx within the SiNW array—rather than a simple core–shell arrangement—maximized the ion-accessible interface, boosting capacitance to 328.6 mF·cm^−2^ (retaining 79.05% over 7000 cycles) and enabling a solid-state asymmetric device with an areal energy density of 0.021 mWh·cm^−2^ [157]. Parallel to inorganic phases, conductive polymer strategies utilize the porous network as a stress-buffering host to mitigate volume expansion and structural degradation. Siddiq et al. confined in situ polymerized polyaniline (PANI) within magnesiothermically reduced mesoporous silicon (mSi); the confinement effect resulted in a specific capacitance of ~214.45 F·g^−1^—a drastic increase from the ~19.85 F·g^−1^ of bare mSi—while maintaining 100% retention after 1000 cycles [158]. This confirms that the mesoporous skeleton essentially “digests” the polymer’s volume effect, a principle echoed in broader micro-device engineering where reinforcing conductive polymer skeletons (e.g., PPy-CNT) is necessary to suppress structural pulverization and sustain long-term cycling (e.g., 79% retention over 10,000 cycles) [159].

For on-chip devices, a hierarchical design comprising “pore skeleton + conductive interlayer + polymer active layer” is equally valid. Zhang et al. demonstrated this on silicon nanowire (SiNW) arrays by spin-coating high-conductivity PEDOT:PSS followed by the electrodeposition of PANI nanofibers. The resulting electrode achieved an areal capacitance of ~301.71 mF·cm^−2^ at 1 mA·cm^−2^, vastly outperforming the control group without the PEDOT:PSS interlayer (~10.18 mF·cm^−2^). Although based on SiNWs, the mechanism applies directly to PSi: when utilizing active layers deep within porous structures, continuous electrical contact is often prioritized over geometric surface area [160]. In summary, recent progress in PSi supercapacitors follows two complementary tracks: (1) EDLC Engineering, utilizing conformal conductive/barrier layers to transform PSi into integrated 3D electrodes, balancing mF·cm^−2^-level capacitance with long-term stability; and (2) Modular Pseudocapacitance, employing active phases to introduce reversible Faradaic processes while serving as passivation layers, thereby enhancing effective capacitance and device usability without sacrificing manufacturability.

To clearly define the application scope of porous silicon in energy storage, it is essential to map its performance against incumbent materials like graphite and activated carbon. While PSi anodes theoretically offer order-of-magnitude improvements in specific capacity, practical implementation requires balancing gravimetric gains against volumetric constraints caused by porosity. Similarly, for supercapacitors, PSi distinguishes itself in high-frequency, on-chip power delivery rather than bulk energy storage. A quantitative comparison of these performance metrics, including energy and power densities, is summarized in Table 3.

### 5.3. Emerging Energy Applications

#### 5.3.1. Porous Silicon as a “Chemical Hydrogen Carrier”: From Reversible Adsorption to On-Demand Hydrolysis

While electrochemical energy storage (LIBs and supercapacitors) represents the most mature application domain for porous silicon (PSi), the material’s intrinsic characteristics—specifically its immense specific surface area, hydrogen-terminated surfaces, and metastable nanoscale skeleton—open distinct avenues for “energy harvesting” and “chemical energy conversion.” Unlike the reversible ion-insertion mechanisms discussed in previous sections, where structural stability is paramount, emerging applications in hydrogen carriers and nanoenergetic systems actively exploit the controlled reactivity of the silicon matrix. In these scenarios, the PSi framework functions not merely as a passive host, but as an active high-energy reagent. This section examines how the precise engineering of pore morphology and interface chemistry allows PSi to serve as a tunable “chemical reservoir,” facilitating either the on-demand generation of hydrogen via hydrolysis or the rapid, exothermic release of energy in on-chip pyrotechnic devices.

In porous silicon (PSi) systems, “hydrogen storage” is generally approached via two distinct pathways: physical adsorption/desorption mediated by the pore structure (reversible storage) and chemical generation via the reaction of the silicon skeleton (or H-terminated surfaces) with water or alkaline media (chemical hydrogen storage via hydrolysis/oxidation). The former is fundamentally limited by the weak binding energy of H_2_ on silicon pore walls at room temperature, resulting in modest capacities. For instance, Muduli et al. systematically evaluated the reversible hydrogen storage of nanostructured ball-milled PSi under pressurized conditions. They reported a maximum adsorption of approximately 665 μmol g^−1^ (≈0.101 wt%) at 20 bar and 120 °C. While extending the window to 70 bar and 500 °C increased adsorption to ≈0.119 wt%, these values remain insufficient for high-density storage requirements, suggesting that reliance on physical adsorption alone is challenging for practical applications [161].

In contrast, the “on-demand chemical generation” pathway does not pursue high-pressure reversibility but rather exploits the high specific surface area and surface termination (Si–H/defects) of porous silicon to accelerate the reaction between Si and water (or alkaline media). This approach transforms “oxidative instability” into an “engineered hydrogen release channel,” targeting the theoretical yield limit of ~1600 mL g^−1^ Si. A definitive example of this “programmable rate” concept is the “Salty Silicon” developed by Martell et al. via salt-assisted (NaCl/KCl) aluminothermic reduction. The resulting p-Si, obtained with an ~83% yield at 850 °C, functions as a packageable unit capable of generating 1476 ± 51 mL H_2_ g^−1^ Si in various aqueous media, with the majority of release occurring within a ~5 min timescale [162].

To prove that chemical release is not synonymous with “uncontrolled corrosion,” Kobayashi et al. demonstrated that reaction kinetics are strictly governed by interfacial chemistry (pH). They established that in pH 8.0 water, Si nanopowder generates ~55 mL g^−1^ in 60 min, yet the initial rate (0–5 min) is ~40 times faster than in ultrapure water, confirming the first-order influence of OH- ions [163]. Furthermore, under strong alkaline conditions (pH 13), the reaction shifts to a high-throughput mode, achieving a rate of 580 mL min^−1^ g^−1^ and a total yield of 1.44 L g^−1^. Moving beyond extrinsic pH control, recent “local chemical field” engineering has pushed kinetics closer to theoretical limits. Such micro-environment control parallels pulsed-laser synthesized single-atom catalysts [164], where confinement dictates reactivity. A study introduced a Si/CaH_2_ composite via ball milling to create a “local high-concentration alkaline field” that promotes continuous hydrolysis; this system achieved a yield of 1216 mL g^−1^ in just 10 min in deionized water, corresponding to a 95.5% conversion rate and an apparent kinetic rate of 225 mL (s·g)^−1^ [165].

Finally, regarding morphological control, Mussabek et al. provided quantitative evidence linking particle size to “programmable kinetics”. Their study on hydrogenated PSi nanopowders revealed that while increasing alkali concentration boosts rates, physical restructuring is equally potent: reducing particle size from the ~100 μm–1 mm range to the 50–200 nm range via grinding enhanced reaction kinetics by approximately 2-fold [54]. This result solidifies the causal link between “structure size—mass transport/oxide evolution—hydrogen generation,” confirming that morphology itself is a tunable variable for regulating energy release.

#### 5.3.2. Porous Silicon Nanoenergetic Systems: From Energy Release Efficiency to On-Chip Micro-Ignition

Composite systems formed by PSi and strong oxidizers (e.g., perchlorates) are categorized as nanoenergetic materials. From a materials science perspective, their superiority stems from two factors: the pore volume and specific surface area of PSi provide a nanoscale mixing interface for the oxidizer, allowing the reaction front to propagate rapidly at the microscale; and the inherent compatibility with silicon processing enables the integration of “energy release” as an addressable on-chip function (e.g., micro-ignition, micro-propulsion, or one-time security triggers).

In terms of reaction characterization, Apaza Quispe et al. conducted multi-modal diagnostics on PSi/perchlorate devices, combining emission spectroscopy (300–1000 nm), acoustic spectrometry (0–500 Hz), and high-speed imaging. Their results correlated output intensity with structural parameters, identifying a rapid energy release event lasting approximately 140–335 ms under optimized conditions. Thermal radiation characteristics suggested combustion temperatures on the order of ~1600 °C, while acoustic signals were concentrated in the 20–380 Hz range, providing quantifiable in situ readouts for device-level deflagration [166].

**Figure 15:**
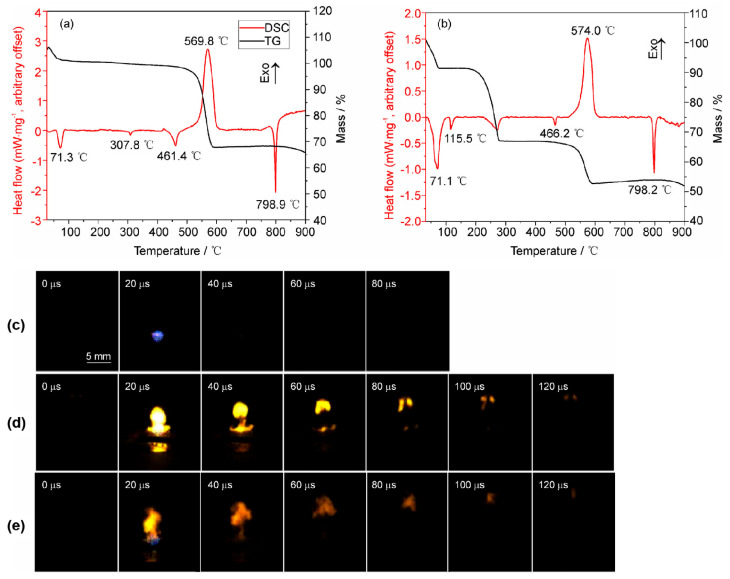
Stabilization of nanoenergetic systems against humidity. (**a**) Thermal analysis (DSC/TG) of fresh silicon nanowire-based energetic films; (**b**) DSC/TG curves for treated films after moisture exposure, where the solid and dashed lines represent the heat flow (DSC) and mass change (TG) respectively, confirming a preserved exothermic peak at approximately 570 °C; (**c**) high-speed imaging sequence of an untreated sample failing to sustain ignition after exposure to 98% relative humidity; (**d**,**e**) high-speed imaging of surface-modified samples demonstrating robust deflagration and flame propagation despite humidity exposure, highlighting the effectiveness of the encapsulation layer [167].

However, the transition from laboratory demonstration to viable devices is constrained by storage stability and environmental adaptability, particularly performance degradation due to oxidizer hygroscopicity. Yang et al. addressed this in silicon nanowire array/perchlorate systems, providing a direct comparison under high humidity (25 °C, 98% RH, 12 h). The surface-modified samples showed a mass increase of only 8.7 mg, compared to 50 mg for untreated controls. Figure 15 provides comprehensive multi-modal evidence for this stabilization. Thermally, DSC/TG analysis confirms that the fundamental energetic potential is preserved despite modification, with both fresh and treated films exhibiting a dominant exothermic reaction peak at approximately ~570 °C (Figure 15a,b). Phenomenologically, high-speed imaging reveals the critical impact of hydration on dynamic performance. While the moisture-exposed untreated sample fails to sustain ignition—manifesting as only a transient, weak discharge visible at 20 μs that extinguishes completely before 40 μs (Figure 15c)—the surface-modified systems retain robust energetic potency. As shown in the replica experiments in Figure 15d,e, even after severe humidity exposure, these samples undergo violent deflagration characterized by rapid flame propagation that sustains high intensity throughout the entire 120 μs observation window. This sharp contrast highlights the inherent contradiction between “pore structures favoring fast reaction” and “environmental moisture inducing failure,” necessitating that interface encapsulation be treated as a primary design variable [167].

Furthermore, device engineering must address the high-risk attribute of “sensitiveness.” Plummer et al. performed standardized sensitivity characterization of PSi nanoenergetic films, reporting extremely low thresholds for activation: impact energy < 4.9 J, friction force < 5 N, and electrostatic discharge (ESD) energy < 45 mJ [168]. These limits dictate that strict safety assessments and isolation strategies are mandatory for engineering implementation. regarding on-chip self-sustained combustion, Piekiel and Morris integrated PSi energetic materials into wafer-scale structures to investigate geometric constraints on propagation. They achieved stable on-chip combustion in micro-machined channels (widths/depths of tens to hundreds of microns), reporting propagation velocities up to 4.6 m s^−1^ and the ability to sustain propagation through 90° turns [169]. This establishes a material–structure integration paradigm for “routable energy release paths”.

In summary, the role of PSi in emerging energy applications is not merely parallel to electrochemical storage but serves as a platform connecting “programmable surface chemistry,” “pore-structure-mediated mass transport,” and “device integration.” In hydrogen applications, it functions as a chemical carrier where kinetics are tuned by structure but limited by oxidation sensitivity; in nanoenergetics, its core advantages are integrability and diagnostic capability, with hygroscopic stability and sensitivity constituting the primary engineering thresholds.

## 6. Applications in Microelectronics and MEMS

### 6.1. RF Passives: Substrate Isolation and Oxidized Porous Silicon

The “substrate dilemma” in silicon RF remains a persistent limitation for integrated passives, stemming from the fact that while silicon is the workhorse of microelectronics, its semiconducting nature (resistivity ρ ≈ 10–20 Ω⋅cm) and high permittivity (ϵr ≈ 11.9) allow electromagnetic fields to penetrate deeply into the bulk. This “substrate participation” introduces displacement currents and eddy current losses that standard thin-film oxides (<1–2 μm) cannot fully suppress. In this context, Oxidized Porous Silicon (OPS) emerges not as an exotic material, but as an in situ processable solution that re-engineers the electromagnetic boundary conditions. By creating isolation layers with micrometer-scale thickness and low ϵr, OPS effectively “pushes” high-frequency electric fields away from the lossy handle, providing a third viable isolation strategy alongside High-Resistivity Silicon (HR-Si) and SOI.

Mechanistically, the efficacy of this thick isolation is governed by the reduction in conductor-substrate equivalent capacitance. Park et al. validated this principle using air-bridge structures formed via anodic porosification and subsequent oxidation, achieving oxide thicknesses up to 10 μm. Their comparative analysis revealed that the OPS-based air-bridge CPW reduced insertion loss by approximately 2 dB at 10 GHz compared to standard OPS-on-Si structures (which exhibited ~3 dB loss), directly confirming that the combination of low-k properties and physical thickness is decisive in mitigating substrate loss channels [170].

Wafer-scale validation and device gain have been successfully demonstrated by positioning OPS as a thick isolation layer. In a representative study, Nam et al. fabricated a 25 μm thick SiO_2_ surface layer on a 6-inch silicon wafer via the porous-silicon/oxidation route; on this substrate, a 50 Ω coplanar waveguide (CPW) exhibited an insertion loss of only 0.03 dB·mm^−1^ at 4 GHz. More significantly, the suppression of substrate eddy currents allowed on-chip inductors in the same process to achieve a maximum quality factor (Qmax) of 120, illustrating that thick low-k dielectrics can simultaneously improve transmission line attenuation and inductive energy storage efficiency. Extending this to the circuit level, Fang et al. compared OPS with HR-Si for planar passives, reporting that a planar low-pass filter on OPS achieved a −3 dB bandwidth of 2.9 GHz with a mid-band insertion loss of 0.87 dB at 500 MHz, while simulation indicated inductor quality factors could exceed 20 under these configurations [171].

**Figure 16:**
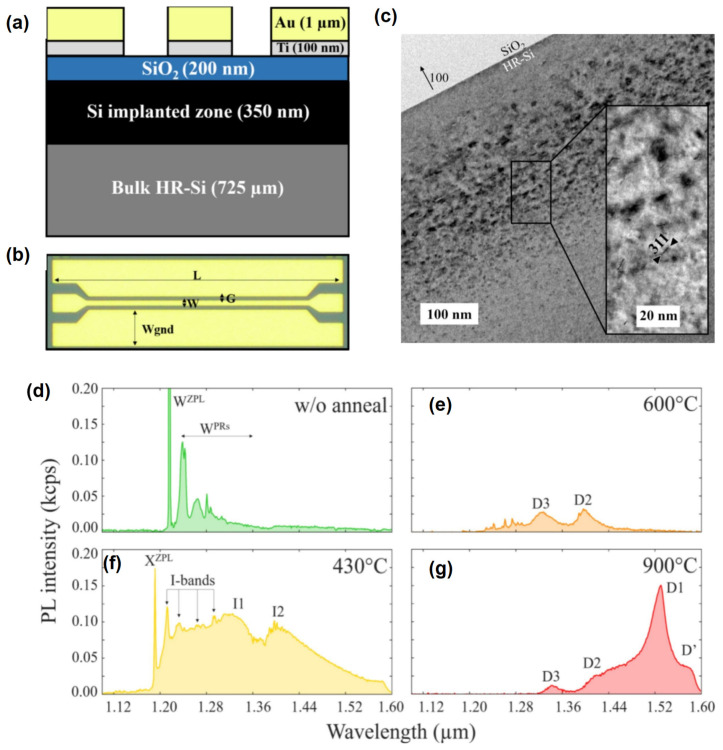
Localized trap-rich substrates for RF isolation. (**a**) Schematic cross-section showing the integration of a Coplanar Waveguide (CPW) on a silicon-implanted zone, and (**b**) the corresponding top-view layout illustrating signal and ground dimensions. (**c**) TEM imaging reveals a dense network of crystallographic defects at the interface. Photoluminescence spectra are presented for (**d**) the as-implanted sample without annealing, (**e**) the sample annealed at 600 °C, (**f**) the sample annealed at 430 °C, and (**g**) the sample annealed at 900 °C. These spectra confirm that the defects act as thermally stable carrier traps, which are essential for suppressing substrate losses [174].

From a fabrication perspective, OPS serves as a platform for manufacturable thick dielectrics, transforming stress-limited thermal growth into a controllable “porosification + step oxidation” sequence. Molinero et al. established a parametric gateway for this approach by extracting material parameters from simple coplanar lines, linking material synthesis directly to RF interconnect modeling rather than just material characterization [172]. Concurrently, recent engineering efforts have addressed wafer-scale uniformity and industrial compatibility. Shams et al. demonstrated the feasibility of uniform porosification on 300 mm wafers—even on standard resistivity silicon—by utilizing backside conductive grids to distribute current [173]. Similarly, Perrosé et al. reported FD-SOI-compatible low-loss RF substrates achieved via localized modification, utilizing silicon implantation to engineer a trap-rich isolating layer. Figure 16 provides the structural and physical validation of this approach: the device architecture (Figure 16a,b) features a coplanar waveguide positioned directly atop a 350 nm thick silicon implanted zone. Cross-sectional TEM imaging (Figure 16c) reveals the microscopic origin of the high resistivity, showing a dense network of interstitial defects and (311) rod-like defects located beneath the thermal oxide. Crucially, photoluminescence spectroscopy (Figure 16d–g) tracks the evolution of these trap states, demonstrating that while the defect signatures shift (e.g., from vacancy-related W/X centers to dislocation-related D-lines) upon annealing up to 900 °C, the deep-level traps required to suppress substrate losses are robustly preserved, highlighting the industry’s sustained demand for localized, thermally stable substrate engineering [174].

Dielectric properties at high-frequency and mm-wave bands become increasingly critical as operating frequencies scale up, necessitating the minimization of effective permittivity (ϵ_eff_) and loss tangent (tanδ). Sarafis and Nassiopoulou quantified this by characterizing CPWs on a 150 μm thick porous silicon layer (76% porosity) in the 140–210 GHz range, extracting an effective relative permittivity of 3.12 ± 0.05 and a loss tangent of 0.023 ± 0.005. These superior dielectric parameters yielded an attenuation as low as ~1 dB·mm^−1^ at 210 GHz and a CPW quality factor of ~30, comparable to high-end substrates like quartz [175]. Furthermore, local substrate engineering targeting specific circuit areas has shown that porous regions can be selectively integrated to decouple RF fields. Issa et al. reported attenuation values of 0.35 dB·mm^−1^ at 60 GHz and 0.55 dB·mm^−1^ at 110 GHz on locally grown porous silicon [176]. Similarly, Belaroussi et al. utilized mesoporous substrates to fabricate a 60 GHz bandpass filter with a remarkably low insertion loss of ~0.5 dB and return loss of ~23 dB, highlighting the competitive advantage of porous substrate engineering when prioritizing CMOS compatibility [177].

### 6.2. Gettering Effects: Impurity Removal in Silicon Wafer Processing

In the context of silicon device fabrication, impurity sequestration via high-surface-area trap layers addresses the persistent issue of metallic impurities (e.g., Fe, Cu, Ni, Co) that exist as deep-level recombination centers and degrade minority carrier lifetime (τ_eff_). Consequently, porous silicon (PSi) is valued not merely as a material phase but as an in situ constructible sink where the abundance of internal surfaces, structural defects, and tunable chemical states creates a high density of low-chemical-potential sites. Mechanistically, this process relies on optimizing the capture kinetics at the extrinsic sink, a step that PSi facilitates by serving as a scalable scaffold to amplify segregation efficiency, particularly when coupled with functional layers for advanced integration.

Addressing the constraints of modern back-end-of-line (BEOL) processes, low-thermal-budget strategies utilizing hybrid zeolite/PSi sinks have emerged to bypass the high temperatures (>850 °C) required by traditional phosphorus diffusion. Medfai et al. demonstrated this route by depositing a Heulandite-Na (HEU-Na) zeolite layer onto a PSi substrate at only 350 °C, creating a synergistic architecture where PSi facilitates mass transport and the zeolite ensures selective sequestration. Quantitative μW-PCD analysis revealed a dramatic enhancement in minority carrier lifetime from 1.44 μs to 30.68 μs (~21-fold increase), accompanied by an increase in diffusion length from 103.27 μm to 324.16 μm and a substantial recovery in Hall mobility from 209.49 to 732.93 cm^2^ V^−1^ s^−1^. This effectively repositions PSi from a high-temperature sacrificial layer to a versatile platform for low-temperature extrinsic gettering [178].

Parallel to mesoporous layers, nanostructured silicon acting as sacrificial sinks provides a geometrically enhanced alternative for high-temperature processing steps. Mannai et al. (2025) utilized silver-assisted chemical etching to fabricate silicon nanowire (SiNW) layers, utilizing them to capture impurities during N_2_ annealing before sacrificial removal. Optimization studies identified 850 °C as the ideal window, yielding a lifetime recovery to 19 μs and an improvement in resistivity from 5.5 to 1.9 Ω·cm with mobility doubling from 122 to 253 cm^2^ V^−1^ s^−1^. Notably, the authors cautioned that exceeding this window (e.g., 900 °C) negates benefits due to defect generation, confirming that the efficacy of the method relies on balancing impurity diffusivity with the thermal stability of the nanostructured scaffold [179].

In scalable manufacturing, achieving process loop closure via removal and surface reformation is decisive, as the incomplete removal of the getter layer can introduce secondary contamination. A study highlighted this critical threshold, demonstrating that replacing standard thermal oxidation/etching with a wet oxidation process significantly improves final wafer quality. This optimized protocol reduced the saturation current density (J0) by approximately 27.0%, improved the apparent effective lifetime by 26.3%, and enhanced the bulk lifetime by 6–14%. These findings underscore that for PSi-based strategies, the “clean removal” of the high-surface-area sink is as critical as the gettering efficiency itself [180].

**Figure 17:**
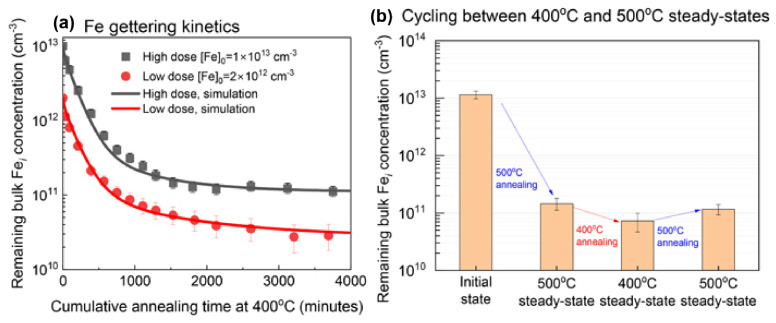
Mechanism of segregation-driven Fe gettering. (**a**) The exponential decay of bulk interstitial iron at 400 °C matches diffusion-limited simulations. (**b**) Reversibility tests show iron concentration oscillating with temperature, confirming a segregation-controlled equilibrium rather than irreversible precipitation [181].

The validity of these composite systems is further supported by mechanistic insights into segregation-driven gettering, which suggest that low-temperature efficiency is governed by diffusion coupling rather than simple precipitation. Le et al. (2024) attributed Fe gettering by ALD-Al_2_O_3_ films to a segregation mechanism limited by the diffusion coupling between the bulk and the film [181]. Figure 17 provides the decisive kinetic fingerprint for this mechanism. As shown in Figure 17a, the remaining bulk interstitial iron (Fei) concentration follows a clear exponential decay at 400 °C, dropping by orders of magnitude (e.g., from an initial 1 × 10^13^ to a steady-state ∼1 × 10^11^ cm^−3^) in excellent agreement with simulation models. Crucially, Figure 17b distinguishes this process from irreversible precipitation by demonstrating temperature-dependent reversibility: cycling the annealing temperature between 400 °C and 500 °C causes the bulk Fe_i_ level to oscillate reproducibly between distinct steady-state values (e.g., ∼8 × 10^10^ vs. ∼1 × 10^11^ cm^−3^). This thermodynamic reversibility confirms that the gettering efficiency is dictated by the interface segregation coefficient. This aligns with earlier findings by Liu and Macdonald, who reported that roughly 50% of dissolved Fe in a 160 μm wafer could be removed via annealing at 425 °C for 30 min. These kinetic frameworks validate the design of “PSi-scaffolded” composite sinks, confirming that under restricted thermal budgets, gettering can be effectively driven by engineered segregation–diffusion coupling [181].

### 6.3. MEMS Integration: PSi as a Sacrificial Layer in Micromachining

Porous silicon (PSi) occupies a distinct niche in Micro-Electro-Mechanical Systems (MEMS) micromachining by functioning as a substrate-native sacrificial layer that can be locally generated via anodization. Unlike deposited films (e.g., SiO_2_), the “sacrificial function” of PSi is implemented in situ: the crystalline substrate itself is converted into a porous phase with tunable density/continuity, enabling either direct removal via wet dissolution/electropolishing to release suspended structures, or thermal reorganization into sealed buried voids. This substrate-native route is particularly attractive when large release gaps, reduced stiction risk, or monocrystalline structural layers are prioritized.

**Figure 18:**
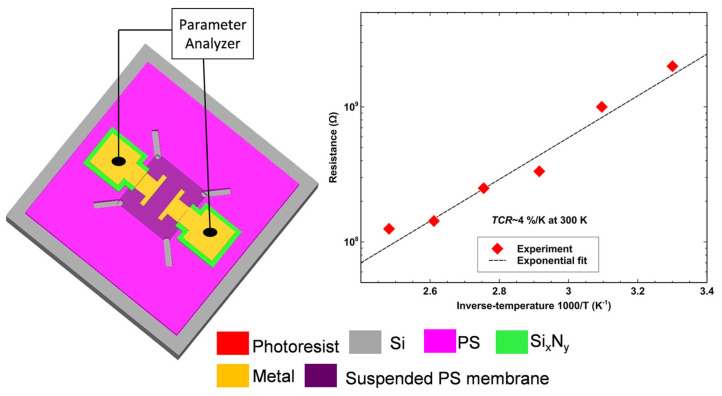
Suspended porous silicon thermal sensor. (**Left**) Schematic of the membrane architecture thermally isolated via suspension arms. (**Right**) Resistance vs. inverse temperature plot confirms Arrhenius behavior, demonstrating the material’s viability for sensitive bolometric applications [186].

In the fabrication of MEMS-grade optical components demanding high mechanical stability, electropolishing-based release strategies have been proven instrumental in defining large-area flat membranes. Afandi and colleagues implemented this strategy in the fabrication of Long-Wave Infrared (LWIR) porous silicon Fabry-Pérot filters, where a multilayer “top mirror” acts as a suspended structure over an air cavity. Their work successfully demonstrated the release of membranes with lateral dimensions ranging from 300 × 300 μm^2^ to 600 × 600 μm^2^, illustrating that PSi-based release technologies are compatible with device-level platforms rather than limited to nanoscale films [182]. Analogous to piezoelectric structural sensing [183,184], these suspended membranes enable precise physical monitoring. This capability was further corroborated by the recent work of Zhang et al. (2025), who utilized porous silicon with 68% porosity as a thermal isolation layer to fabricate a MEMS Pirani vacuum gauge; the device maintained excellent mechanical integrity under transient pressure shocks up to 4 × 10^5^ Pa, while exhibiting a thermal conductivity as low as 3.5 W/(m·K) [185]. The recent study by Sharma et al. (2024) provides robust data support for this advantage; as depicted in the device schematic in Figure 18, they successfully fabricated a suspended PS membrane bridged by supporting arms and electrically probed via metal pads [186]. Experimental characterization revealed a strict exponential dependence of resistance on inverse temperature (Arrhenius behavior), and by designing a heterogeneous stack structure combining low (48%) and high (80%) porosities, they not only achieved a high TCR of 4.4%⋅K^−1^, but also successfully suppressed the 1/f noise constant to a level of 4 × 10^13^, validating the feasibility of this strategy in high-performance sensing applications [186].

The use of PSi is a transformable precursor to buried cavities, a concept epitomized by the Advanced Porous Silicon Membrane (APSM) process. As described in industry reports, this sequence—comprising local porosification, annealing-induced rearrangement, and epitaxial sealing—yields a monocrystalline silicon membrane over a defined vacuum cavity, fundamentally distinguishing it from the isotropic under-etching of sacrificial oxides [187]. This distinction was rigorously validated by Gong et al. (2024), who applied a “Silicon Migration Seal” (SMS) technique—functionally analogous to APSM—to fabricate vacuum-encapsulated MEMS resonators [188]. Their process achieved a fabrication yield exceeding 95% and maintained a stable cavity pressure below a few hundred Pascals, proving that the annealing-induced rearrangement delivers superior hermeticity and structural integrity compared to traditional release methods [188]. The transition of this technology into high-volume manufacturing is evidenced by its application in low-cost far-infrared focal plane arrays, where the APSM process forms the structural foundation for thermodiode readout junctions. Furthermore, recent disclosures indicate that APSM-derived membranes serve as modular building blocks for complex architectures; for instance, process flows integrating membranes with stress-optimized dielectric stacks and access holes demonstrate how PSi-enabled substrates can initiate downstream MEMS structuring [189]. Demonstrating this modular versatility, Chen et al. (2024) successfully integrated piezoelectric Sc-doped AlN thin films onto Silicon-on-Nothing (SON) resonating membranes [190]. By utilizing the buried cavity to decouple the resonator from the substrate, they achieved precise low-vacuum sensing capabilities (down to ~69 mbar), validating that such transformable substrates can effectively support stress-sensitive functional layers [190].

Overall, recent examples support a consistent conclusion: PSi sacrificial-layer micromachining is less about substituting SiO_2_ and more about enabling “substrate-native cavity engineering”. Whether providing a tunable layer that supports large-area suspended structures (hundreds of micrometers) via electropolishing, or acting as a precursor to epitaxially sealed voids for wafer-level integration, PSi aligns naturally with the demands of high-performance MEMS platforms.

### 6.4. On-Chip Passives: High-Density Trench Capacitors

High-density trench capacitors represent the quintessential strategy for “embedding capacitance area into silicon,” addressing the limitation of planar geometries where areal capacitance is strictly bound by dielectric breakdown limits. By fabricating arrays of deep trenches or macropores within the silicon substrate, the effective electrode area (A_eff_) is amplified by tens to hundreds of times relative to the projected footprint (A). When coupled with thin dielectric layers, this architecture achieves areal capacitance densities far exceeding those of planar MIM or MOS devices. The utility of this approach extends beyond maximizing capacitance-per-area (C/A); it centers on enabling proximal decoupling and transient energy buffering near the power distribution network (PDN). By minimizing the physical distance to the load, parasitic inductance and resistance are suppressed, satisfying the demands of 10–100 MHz switching power supplies and high-speed digital/RF systems for moderate capacitance with low Equivalent Series Inductance (ESL) and Resistance (ESR) [191,192].

**Figure 19:**
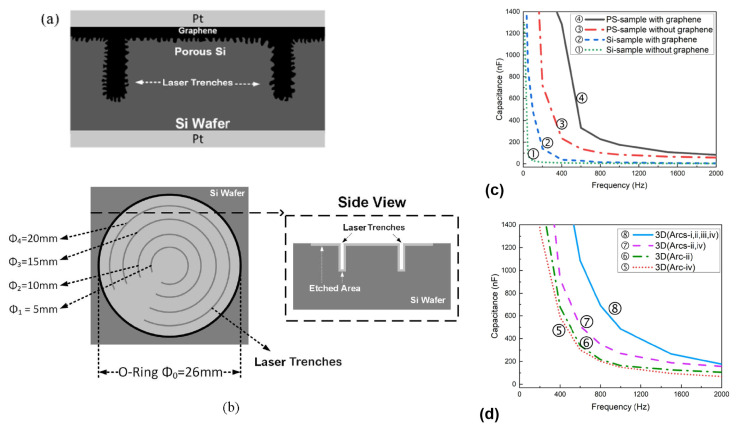
Hybrid laser-electrochemical 3D trench capacitors. (**a**,**b**) Laser scribing defines the macro-geometry, serving as initiation sites for electrochemical pore growth. (**c**,**d**) Capacitance measurements show that graphene-enhanced porous structures significantly outperform planar controls, with capacity scaling predictably with trench complexity [194].

In the context of porous silicon materials, the relevance of these devices lies in the structural formation mechanism. While Deep Reactive Ion Etching (DRIE) is the standard industrial tool, electrochemical micromachining—fundamentally homologous to PSi formation—enables the fabrication of significantly higher aspect ratio and denser 3D silicon skeletons. Strambini et al. demonstrated this capability by fabricating high-density trench arrays with aspect ratios up to 100, subsequently depositing MIM nanolaminates (e.g., TiN/Al_2_O_3_/TiN) via Atomic Layer Deposition (ALD). They reported areal capacitance densities reaching the 1 µF/mm^2^ regime, with operating voltages up to 16 V and frequencies up to ~70 kHz, explicitly positioning electrochemical microstructuring as a route to “on-chip energy storage” [193]. Further exploring process scalability, Lin et al. proposed a hybrid architecture integrating electrochemical 3D etching into laser-scribed trench sidewalls. Figure 19 illustrates this “macro-micro” synergy: laser ablation defines the primary trench geometry (Figure 19b), which subsequently serves as the initiation site for lateral porous silicon growth, effectively expanding the surface area into the silicon bulk (Figure 19a). Crucially, the authors addressed the high series resistance of the porous skeleton by embedding graphene into the surface pores. The impact of this interface engineering is quantified in Figure 19c: while planar silicon and unmodified PS samples show limited charge storage, the “PS-sample with graphene” exhibits a dramatic capacitance enhancement, achieving values exceeding 1400 nF in the low-frequency regime. Finally, Figure 19d confirms the geometric tunability of the device: the capacitance scales predictably with the complexity of the trench pattern (e.g., number of concentric arcs), demonstrating that the electrochemical expansion is strictly confined by the initial laser patterning and allowing for deterministic capacity design [194].

Complementing this structural evolution is the roadmap for dielectric materials, which has shifted from standard Oxide/Nitride/Oxide (ONO) stacks toward low-resistance electrodes and high-k dielectrics. Roozeboom et al. systematically characterized trench capacitors based on ~30 µm deep macropore arrays coated with ONO and polysilicon electrodes. Their work demonstrated decoupling capacitance densities of ~30 nF/mm^2^ with robust breakdown voltages around ~30 V; reducing dielectric thickness further boosted density to the ~80 nF/mm^2^ range, validating the low ESL/ESR benefits at the system level [191]. However, pushing densities beyond >200 nF/mm^2^ requires overcoming the challenges of high-aspect-ratio deposition [195]. As summarized in ECS Transactions, achieving these targets necessitates thinner dielectrics and high-k/metal gate combinations, where low-temperature (≤400 °C), highly conformal ALD is the critical enabling technology to mitigate roughness-induced breakdown and interfacial oxidation.

From a system-level perspective, industrial adoption is increasingly driven by the need for robust in-package decoupling. As power converter frequencies rise, the requirement shifts from “bulk capacitance” to “moderate capacitance with ultra-low parasitics.” Silicon trench capacitors (e.g., IPDiA’s PICS series) address this window by employing multi-layer Silicon–Isolation–Silicon (SIS) stacking to engineer ESR/ESL at the device layout level. Reliability assessments have further validated these devices for operation up to 225 °C, confirming their suitability for harsh environments. The consensus spanning academia and industry is that trench capacitors are bridging the gap between microelectronic filtering and energy buffering. Within this scope, the combination of 3D convertible silicon skeletons provided by PSi technologies—offering superior aspect ratios and design flexibility—with advanced ALD stacks continues to drive the evolution toward higher areal density and reliability.

In summary, the integration of porous silicon into microelectronic workflows relies on its ability to offer distinct performance advantages over standard industrial processes. Whether serving as a thick, low-loss dielectric for RF isolation, a high-efficiency sink for impurity gettering, or a substrate-native sacrificial layer for MEMS, PSi must compete with established technologies like High-Resistivity Silicon (HR-Si) and deposited thin films. A comparative benchmarking of these applications, highlighting the specific pros and cons of PSi against incumbent technologies, is presented in Table 4.

## 7. Rational Design Guidelines: Balancing Trade-Offs

To successfully translate the diverse capabilities of porous silicon (PSi) from laboratory demonstrations to functional devices, researchers must navigate a complex landscape of material properties where a “one-size-fits-all” morphology does not exist. Instead, the optimal design is strictly defined by the rate-limiting step of the specific application, whether it be mass transport, interfacial kinetics, or structural mechanics. Bridging the gap between fundamental chemistry and engineering constraints requires a holistic consideration of geometric matching, chemical stability, and process integration [79].

From a geometric perspective, the selection of pore diameter (dp) is a functional boundary condition governed primarily by the hydrodynamic radius (Rh) of the target species. In optical biosensing, avoiding the Knudsen diffusion regime is paramount [196]; thus, the pore diameter should ideally exceed three times the molecule’s hydrodynamic radius (dp > 3 Rh). While micropores (<2 nm) offer maximum surface area, they are often inaccessible to large proteins, necessitating open mesopores or macropores to ensure effective analyte infiltration [79]. Conversely, in energy storage applications like Li-ion anodes, the geometric priority shifts to accommodating the ~300% volume expansion of silicon [197]. A hierarchical design is often required to balance this expansion buffering with volumetric energy density, as excessive porosity inevitably leads to low tap density and poor electrical connectivity.

Parallel to geometric constraints, the thermodynamic instability of the native hydride surface dictates the device’s operational lifetime. While the as-anodized Si–H surface is convenient for synthesis, its reactivity leads to zero-point drift in sensors and continuous side reactions in electrolytes. For aqueous and biological operations, decoupling the interface from the silicon skeleton via thermal oxidation (forming Si–O–Si) or hydrosilylation (forming Si–C) is essential to prevent hydrolysis [67]. In electrochemical environments, this challenge evolves into managing the solid electrolyte interphase (SEI); design strategies must focus on “sealing” the external surface—for instance, through carbon coating or atomic layer deposition—to limit SEI formation to a stable outer shell rather than allowing it to penetrate and clog the active porous network [198].

Finally, the fabrication route must align with the system-level architecture and processing constraints. Discrete components, such as battery slurries, benefit from cost-effective, scalable methods like stain etching or powder-based MACE, where particle uniformity takes precedence over placement precision. In contrast, monolithic integration with CMOS demands strict adherence to thermal budgets and contamination protocols [199]. This often necessitates the adoption of vapor-phase etching or dry-removal techniques to circumvent capillary collapse and prevent metal contamination, ensuring that the porous functionality is realized without compromising the yield of surrounding electronic circuits. We summarize these critical structure–property trade-offs and recommended design rules in Table 5, serving as a practical guide for material selection.

## 8. Challenges and Future Perspectives

Despite the demonstrated versatility of porous silicon (PSi), its translation from laboratory coupons to ubiquitous industrial components is constrained by the dichotomy between intrinsic material reactivity and the rigorous demands of large-scale manufacturing [200]. To bridge this gap, future development must move beyond characterizing failure modes to implementing specific pathways to solutions, particularly regarding the material’s metastable interface.

The primary obstacle to industrial reliability is the thermodynamic instability of the native hydride-terminated surface [200], which leads to shelf-life degradation in sensors and uncontrolled electrolyte consumption (SEI thickening) in batteries [65]. To resolve this, the industry is moving from simple monolayer termination toward Hybrid Passivation Architectures [65]. In the context of high-energy-density anodes, a solution lies in “Internal/External Partitioning,” where the internal pore surface is stabilized via thermal carbonization to ensure electrical connectivity, while the external particle surface is hermetically sealed with a dense carbon shell or conductive polymer [72]. This composite architecture effectively decouples the volume-buffering function of the pores from the chemical reactivity of the interface, preventing continuous SEI formation while maintaining high capacity.

Parallel to energy applications, sensing and microelectronic integration require a “Dual-Barrier Strategy” to mitigate zero-point drift without compromising sensitivity. A viable pathway involves reinforcing the initial chemical functionalization (e.g., hydrosilylation) with an ultra-thin (<5 nm) conformal inorganic coating, such as ALD-deposited Al_2_O_3_ or SiO_2_. This inorganic “cage” provides a kinetic barrier against oxidative drift and hydrolysis in harsh environments, effectively locking the sensor’s baseline [67,201]. By combining organic specificity with inorganic robustness, this approach addresses the rigorous stability standards required for commercial diagnostic devices.

Finally, regarding manufacturing scalability, the paradigm must shift from wet-chemical processing to Vapor-Phase Manufacturing Protocols. Traditional liquid-phase etching struggles with wafer-scale uniformity and creates contamination risks incompatible with modern CMOS cleanrooms [202]. Adopting vapor-phase metal-assisted chemical etching allows for the definition of porous structures using standard dry-etching equipment, eliminating surface tension-induced collapse and facilitating monolithic integration. Simultaneously, for cost-sensitive applications, the economic pathway involves establishing “Upcycling Supply Chains”, where photovoltaic kerf loss is converted into porous anode powders. This strategy aligns production with circular economy principles [203,204].

## 9. Conclusions

Porous silicon (PSi) has evolved from a material defined primarily by its formation mechanism into a sophisticated platform driven by programmable morphology and interface engineering. While this review has demonstrated its versatility across optoelectronics, energy storage, and microsystems, the transition from academic demonstration to ubiquitous industrial adoption is currently constrained by inherent material and processing limitations.

Main Challenges:

1. Interfacial Stability: Despite the development of diverse passivation routes—from thermal oxidation to hydrosilylation—the thermodynamic instability of the native hydride surface remains a primary failure mode. This reactivity leads to signal drift in biological sensors and uncontrolled solid electrolyte interphase (SEI) growth in high-energy-density batteries.
2. CMOS Compatibility: The predominant reliance on wet-chemical etching (anodization and liquid-phase MACE) introduces contamination risks and capillary stiction issues. These “wet” processes are often incompatible with the strict contamination protocols and dry-processing standards of modern semiconductor foundries.
3. Scalability: While laboratory-scale synthesis allows for precise morphological control, replicating atomic-level pore uniformity across large-format wafers (e.g., 300 mm) presents significant engineering hurdles for mass production.

Future Research Directions:

To navigate these trade-offs and maximize practical impact, the field must pivot toward the following strategic areas:

1. From “Selective” to “Smart” Sensing: Future sensing technologies must evolve beyond reliance on single-analyte specificity toward multidimensional “smart” recognition. Given that porous silicon sensors generate rich and complex optical fingerprints, the breakthrough lies in their deep integration with advanced computational paradigms. By coupling the material’s optical response with signal optimization frameworks and secure distributed computing architectures, researchers can precisely extract feature information from environmental background noise. Ultimately, ensuring device reliability requires a holistic design that aligns infrastructure resilience with predictive classification algorithms, enabling the robust decoding of multi-component targets in dynamic, real-world environments.
2. Solid-State Energy Integration: As energy storage paradigms shift toward solid-state batteries, the critical bottleneck lies in maintaining a coherent interface between the expanding porous silicon anode and the rigid electrolyte. To bridge this “chemo-mechanical” gap, future PSi architectures must evolve beyond simple porosity toward multi-disciplinary integration. By adopting catalytic regulation principles [139] to optimize interfacial kinetics, and incorporating mechanical toughening and self-healing mechanisms—such as the stress recovery behavior observed in amorphous systems [205] and the strength–plasticity synergy of advanced alloys [206]—researchers can engineer a “resilient” silicon skeleton. This approach ensures that the anode can withstand repeated volume fluctuations without suffering from physical delamination, thereby sustaining stable solid–solid contact.
3. Vapor-Phase Manufacturing: To truly bridge the gap between laboratory synthesis and microelectronic foundries, the manufacturing paradigm must shift from wet-chemical baths to standardized vapor-phase protocols. Traditional liquid-phase etching suffers from surface-tension-induced structural collapse and ionic contamination, rendering it incompatible with modern sub-10 nm CMOS cleanroom standards. Establishing robust, dry etching routes (e.g., gas-phase MACE) is therefore the decisive step to enable the monolithic integration of PSi sensing or RF modules directly onto active circuitry, effectively decoupling morphological control from solvent limitations.
4. Sustainable Upcycling: The commercialization of PSi in energy storage is currently constrained by the prohibitive cost of electronic-grade wafers relative to graphite. A critical pathway for economic viability lies in sustainable upcycling, specifically converting industrial silicon waste—such as photovoltaic kerf loss—into high-performance porous anodes. By adapting scalable synthesis techniques (e.g., powder-based etching) to these low-cost feedstocks, the field can establish a circular material ecosystem, effectively lowering the cost-per-kWh. While material upcycling lowers the hardware cost-per-kWh, the economic viability of future smart sensing networks also depends on the computational cost. Adopting unified metric architectures for AI infrastructure [207] and cost-preserving pricing models [208] will be essential to manage the immense data processing expenses associated with large-scale sensor deployment. However, it is crucial to recognize that establishing a stable circular supply chain is not solely a technical challenge; it equally depends on robust corporate governance to ensure long-term investment returns. For instance, strong stakeholder protection mechanisms [209] and effective employee incentive strategies [210] are vital for maintaining the operational stability of deep-tech battery ventures, constituting the essential “soft infrastructure” for successful technology translation.

## Figures and Tables

**Table 1:** Comparison of Key Performance Metrics for Room-Temperature Gas Sensors.

Sensor Material	Sensitivity (LOD)	Selectivity	Response/Recovery Time	Stability	Power/Integration Cost
Porous Silicon (PSi)	Moderate to High (~ppm to ppb level)	Tunable (High with specific functionalization)	Moderate (10–60 s, diffusion limited)	Moderate (Requires passivation to prevent oxidation)	Low Power/Low Cost (Room Temp; Monolithic CMOS compatible)
Metal Oxides (MOX) (e.g., SnO_2_, ZnO)	High (ppb level)	Low (Broad cross-sensitivity)	Fast (<10 s)	High (Chemically robust)	High Power (Requires heating >200 °C); Low material cost but higher system power cost
Carbon Nanotubes (CNTs)	Very High (Theoretical single molecule)	Low (Requires functionalization)	Fast (Surface interaction)	High (Strong C-C bonds)	Low Power/High Cost (Difficult to align/transfer; expensive synthesis)
Conductive Polymers	Moderate	Moderate (Tailorable chemistry)	Moderate to Slow	Low (Sensitive to humidity/temp degradation)	Low Power/Low Cost (Solution processable)

**Table 2:** Performance Comparison of PSi Biosensors with Competing Technologies.

Platform	Detection Principle	Limit of Detection (LOD)	Label-Free?	Advantages	Limitations
Porous Silicon (PSi)	Optical (Refractive Index)/Electrical	High (~nM to pM; 10^−7^ RIU)	Yes	High Surface Area (3D); CMOS compatible; Low cost	Mass transport limited in deep pores; Stability in bio-fluids
Surface Plasmon Resonance (SPR)	Optical (Plasmonic)	High (~nM; 10^−7^ RIU)	Yes	Industry Gold Standard; Real-time kinetics	High Cost; Bulky optical instrumentation; Planar (2D) binding only
Graphene/CNT FETs	Electrical (Field Effect)	Ultra-High (fM to aM)	Yes	Extreme Sensitivity; Rapid electrical readout	Low Reproducibility; Debye length screening in high-salt buffers
ELISA	Optical (Colorimetric/Fluorescence)	High (pM)	No	High Specificity; Well-established clinical workflow	Labor Intensive; End-point only (no kinetics); Requires labeling

**Table 3:** Comparison of Energy Storage Metrics.

Device Type	Electrode Material	Energy Density (Wh/kg)	Power Density (W/kg)	Cycle Life (Cycles)	Key Characteristic
Li-Ion Battery (Anode)	Porous Silicon (PSi)	High	Moderate	Moderate	High Capacity; buffers volume expansion; lower tap density
		(500–1000+)	(100–1000)	(500–1000)	
	Graphite (Standard)	Moderate	Moderate	High	Standard Stability; limited capacity (372 mAh/g)
		(100–260)	(<1000)	(>2000)	
Supercapacitor	PSi (On-Chip)	Low to Moderate	Very High	Very High	Integrated Power; high frequency response; CMOS compatible
		(<10)	(>10,000)	(>100,000)	
	Activated Carbon	Moderate	High	High	Bulk Storage; low cost; not suitable for on-chip integration
		(5–10)	(500–5000)	(>100,000)	

**Table 4:** Benchmarking PSi in Microelectronics & MEMS vs. Standard Technologies.

Application Domain	Material/Technology	Key Performance Metrics	Pros	Cons
RF Passive Isolation	Oxidized PSi (OPS)	Insertion Loss: Very Low (<0.1 dB/mm) Dielectric Thickness: High (>10–100 µm)	Thick Isolation; Tunable permittivity; CMOS compatible formation	Mechanical fragility if not fully oxidized
	Standard SiO_2_ (Thin Film)	Insertion Loss: Moderate Thickness: Limited (<3 µm)	Standard Process; High breakdown strength	Stress-limited thickness; insufficient for high-freq decoupling
	High-Resistivity Si (HR-Si)	Loss: Low Resistivity: >1 kΩ·cm	Wafer-level solution	Expensive; Prone to parasitic surface conduction (PSC)
MEMS Sacrificial Layer	Porous Silicon	Etch Selectivity: High (>1000:1 vs. Si) Release Rate: Fast	In situ Formation; Enables monocrystalline membranes (APSM)	Requires precise drying (Critical Point Drying) to avoid stiction
	PECVD Oxide/Phosphosilicate Glass	Selectivity: High (vs. Si)	Industry Standard; Easy to deposit	Slow Release in long channels; limited by deposition rate
Gettering	Porous Silicon Layer	Efficiency: High (Segregation coeff > 10^4^)	Low Thermal Budget; High surface area sink	Requires removal step (polishing/etching) after gettering
	Polysilicon Backside	Efficiency: Moderate	Standard; No removal needed	High temperature activation required

**Table 5:** Practical Design Guidelines for Porous Silicon Devices.

Application Domain	Critical Trade-off	Design Rule/Guideline	Recommended Morphology
Optical Biosensing	Sensitivity vs. Kinetics	Match pore size to analyte.	Open Mesopores/Macropores
	(Small pores increase surface area but hinder diffusion)	Ensure pore diameter is >3× the target molecule size to avoid Knudsen diffusion limits.	(Flow-through membranes preferred)
Gas/Chemical Sensing	Response vs. Recovery	Passivate to prevent drift.	Hierarchical Pores
	(High energy binding sites cause hysteresis)	Use hydrophobic modification (e.g., hydrosilylation) to suppress water interference; avoid unconnected micropores.	(Large channels for transport + small pores for sensing)
Li-Ion Anodes	Expansion vs. Density	Internal/External Partitioning.	Micron-sized Porous Particles
	(High porosity buffers volume but lowers volumetric energy)	Use internal porosity to accommodate expansion, but seal the external surface (e.g., C-coating) to limit SEI formation.	(Tap density optimized)
RF/Microelectronics	Isolation vs. Strength	Maximize oxidation depth.	High-Porosity Mesopores
	(High porosity lowers permittivity but weakens structure)	Convert fully to thick porous oxide (OPS) to remove the lossy silicon skeleton while maintaining structural pillars.	(Fully Oxidized)
MEMS Sacrificial Layers	Release Rate vs. Stiffness	Control capillary forces.	Bilayer Stacks
		Use high-porosity layers for fast release, but employ critical point drying (CPD) or stiffeners to prevent stiction.	(Low porosity support + High porosity release layer)

## Data Availability

No new data were created or analyzed in this study.
